# Mechanisms of high-glucose-induced mitochondrial damage and glycolipid accumulation in largemouth bass

**DOI:** 10.1186/s40104-025-01261-2

**Published:** 2025-10-15

**Authors:** Zhihong Liao, Xuanshu He, Xingyu Gu, Tao Ye, Anqi Chen, Yucai Guo, Wei Zhao, Jin Niu

**Affiliations:** State Key Laboratory of Biocontrol, Guangdong Provincial Key Laboratory for Aquatic Economic Animals and Southern Marine Science and Engineering Guangdong Laboratory (Zhuhai), School of Life Sciences, Sun Yat-Sen University, Haizhu District, No. 135, Xingang West Road, Haizhu District, Guangzhou, China

**Keywords:** Largemouth bass, Mitochondrial function, Pink1/Parkin, Sirt1

## Abstract

**Background:**

The carnivorous fish, largemouth bass (*Micropterus salmoides*), has difficulty metabolizing dietary carbohydrates, frequently resulting in issues with energy metabolism and fatty liver disease. Nevertheless, the molecular mechanisms involved are still not fully understood.

**Results:**

The results of high-carbohydrate (HC) diets and high-glucose (HG) treatments in largemouth bass hepatocytes showed that high-glucose causes liver damage and glycolipid accumulation. High-glucose promoted the lipogenesis process by activating AMPK/ACC/SREBP-1 pathway and reduced bile acid synthesis by downregulating cholesterol 7-hydroxylase (*cyp7a1*) and sterol 12-hydroxylase (*cyp8b1*). Concurrently, HG treatments also caused mitochondrial fission and damage by increasing the expression of dynamin-related protein 1 (Drp1), leading to impaired mitochondria accumulation and mitochondria-dependent apoptosis via the p38 MAPK/Bcl-2/Casp3 pathway. Additionally, HG treatments decreased Sirt1 expression and relocated it from the nucleus to the cytoplasm, where it interacts with autophagosomes and lysosomes, inhibiting Pink1/Parkin-mediated mitophagy. This also led to the cytoplasmic translocation of Pink1 and its co-localization with Sirt1, indicating that Sirt1 regulates high glucose-induced metabolic stress by inhibiting the Pink1/Parkin mitophagy pathway.

**Conclusion:**

In summary, HG treatment induces mitochondrial damage and glycolipid accumulation in largemouth bass through mechanisms involving AMPK/SREBP1/ACC1-mediated lipogenesis, bile acid metabolism, Sirt-mediated mitophagy, and p38 MAPK/Bcl-2/Casp3-activated apoptosis.

**Graphical Abstract:**

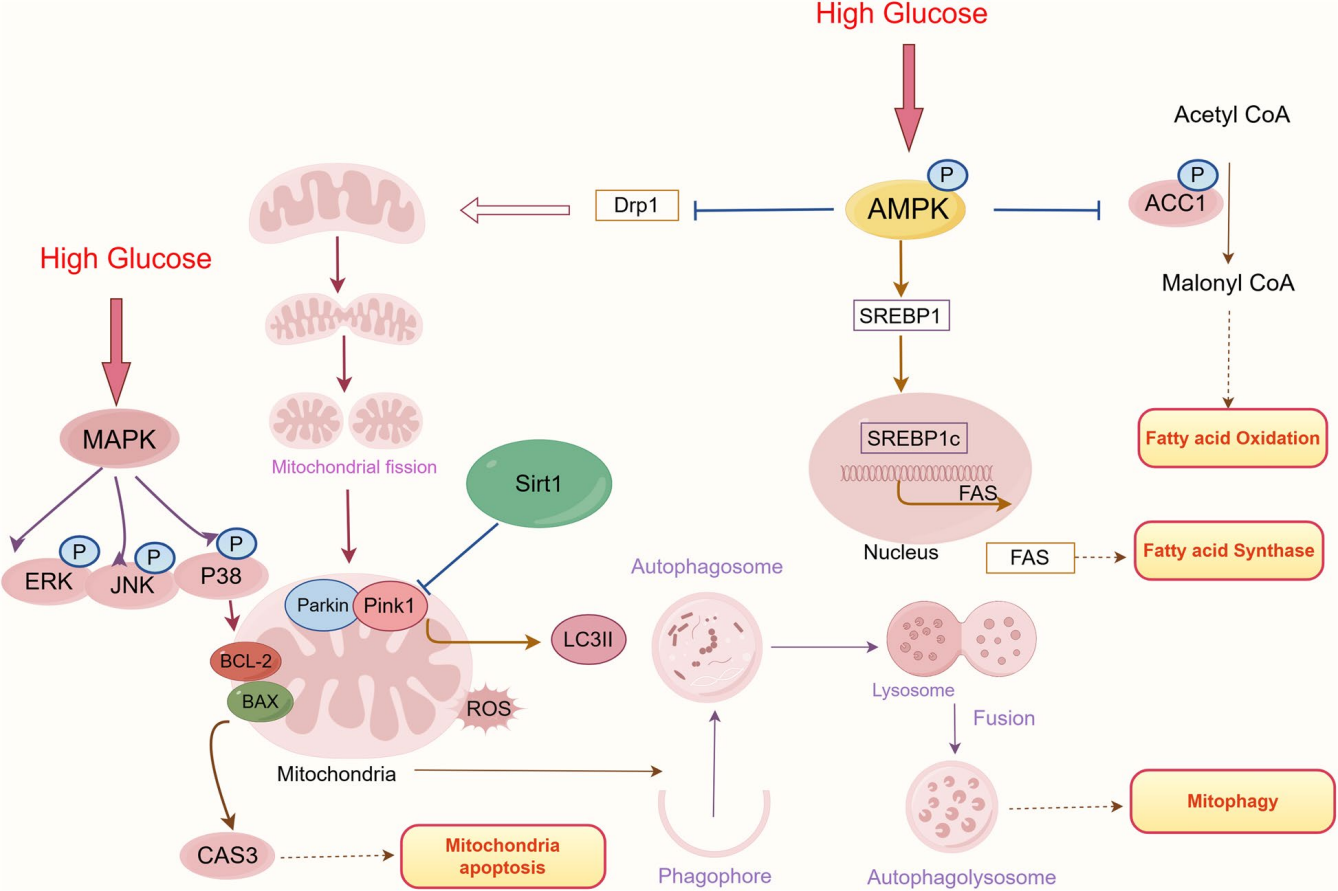

## Background

In recent years, aquacultural production of largemouth bass (*Micropterus salmoides*) in China has rapidly expanded in parallel with the development and optimization of compound feed. By 2025, Chinese aquaculture production of largemouth bass is predicted to reach 1,000 thousand tons [1]. Multiple studies have demonstrated that an excessive carbohydrate content (exceeding 10%) in the formula feed of largemouth bass can lead to liver damage [2–6]. Additionally, high-carbohydrate diets have been shown to induce vacuolization of liver parenchymal cells and reduce survival in largemouth bass, which may be correlated to increased liver damage [7, 8]. Therefore, low-carbohydrate feed has become the predominant choice within the largemouth bass industry [9]. However, the excessive use of fish meal in low-carbohydrate feed leads to elevated costs and environmental concerns, significantly impeding the industry's development. 

Excessive lipid and glycogen accumulation in the liver has been identified as a critical factor contributing to growth retardation and hepatic damage in fish subjected to a high-carbohydrate diet [10, 11]. Hepatic fat accumulation generally arises from an imbalance between lipogenesis and lipolysis, mediated by various transcriptional regulators and enzymatic activities involved in hepatic lipid homeostasis [12]. Xie et al. [13] found that high-carbohydrate diets promote greater fat accumulation in Nile tilapia (*Oreochromis niloticus*) than high-fat diets. In hybrid grouper (*Epinephelus fuscoguttatus* ♀ × *E*. *lanceolatus* ♂), a high-carbohydrate diet significantly downregulated adipose triglyceride lipase (a*tgl)* expression, leading to hepatic triglyceride accumulation [14]. The expression levels of fatty acid synthase (*fas*) and acetyl-CoA carboxylase 1 (*acc1*) were significantly up-regulated, resulting in increased plasma cholesterol and total fat content in grass carp fed with high-carbohydrate diets [15]. Furthermore, it was also shown that AMP-activated protein kinase (AMPK) and sterol regulatory element-binding protein 1 (*srebp1*) are both involved in triglyceride accumulation in largemouth bass fed high-carbohydrate diet [16]. To fully clarify the regulatory effect of a high-carbohydrate diet on fatty metabolism, we analyzed the related genes of lipolysis/fatty acid oxidation, lipogenesis, and fatty acid transport.

Within the classic bile acid synthesis pathway FXR-mediated *shp* decreases bile acid biosynthesis by inhibiting *cyp7a1* expression [17]. Concurrently, FXR stimulates FGF-19 in the enterocytes, which subsequently activates fibroblast growth factor receptor 4 (*fgfr4*). This activation triggers the c-JUN and ERK signaling pathways, leading to a reduction in new bile acid synthesis through negative feedback mechanisms [18]. However, research on MAPK signaling pathways in farmed fish remains nascent, with limited understanding of the detailed mechanisms and regulatory functions [19–23]. In Nile tilapia, it has been observed that the NF-κB pathway, rather than the p38 MAPK pathway, is implicated in intestinal inflammation induced by high-carbohydrate diets [24]. In largemouth bass, high-carbohydrate intake may impair spleen immune function through an inflammatory response mediated by the MAPK/FoxO pathway [25]. To elucidate the mechanisms underlying high-carbohydrate-induced lipid metabolic disorders in largemouth bass, this study would concentrate on the MAPK signaling pathway with in vivo and vitro.

In mammals, mitochondrial dysfunction constitutes a major pathogenic factor in metabolic disorders such as insulin resistance and diabetes [26]. Fish, as ectothermic organisms, exhibit unique mitochondrial adaptations that are essential for their survival in diverse aquatic environments [27]. Oxidized fish oils have been demonstrated to induce significant depletion of mitochondrial membrane potential and drastically reduce ATP production in the liver of yellow catfish (*Pelteobagrus fulvidraco*) [28]. Complementary transcriptomic analyses by Prisingkorn et al. [29] revealed that high-fat-high-carbohydrate diets substantially downregulate expression of key mitochondrial biogenesis genes, including PPARγ-coactivator 1α (PGC-1α), establishing a direct molecular link to mitochondrial impairment. Substantiating these findings, Shen et al. [30] documented profound mitochondrial dysfunction in high-glucose cultured tilapia hepatocytes, evidenced by diminished mitochondrial density, coupled with markedly attenuated activities of respiratory chain Complexes II and III. Investigations in blunt snout bream (*Megalobrama amblycephala*) further demonstrated that high-carbohydrate diets disrupt mitochondrial dynamics through dysregulated fission–fusion equilibrium, consequently suppressing oxidative phosphorylation capacity [31]. Therefore, understanding how high-glucose impacts mitochondrial function could provide insights into metabolic disorders in fish and inform dietary strategies to mitigate these effects.

Sirt1, as a central regulator of energy metabolism, forms a synergistic network with the AMPK signaling pathway to maintain mitophagic homeostasis [‌^32^]. Mechanistic studies reveal that Sirt1 activates AMPK through deacetylation modifications, directly promoting autophagosome formation and enhancing mitophagic flux‌ [33]. This regulatory mechanism is particularly critical for eliminating mitochondria damaged by oxidative stress [‌^34^]. The Pink1/Parkin pathway is a core executor of mitophagy and exhibits functional coupling with Sirt1 [35]. The Sirt1-Sirt3 regulatory axis enhances mitochondrial membrane localization stability of Pink1 and optimizes Parkin-mediated ubiquitination, thereby improving the recognition and clearance of dysfunctional mitochondria [36]. Notably, Sirt1 modulates the opening dynamics of mitochondrial permeability transition pores (mPTP), which governs cytochrome c release kinetics and acts as a pivotal checkpoint in metabolic inflammation and apoptotic cascades [37]‌.

Current research on the metabolic effects of high-carbohydrate diets in largemouth bass primarily focuses on animal models (e.g., feed substitution, growth and enzyme activity analysis), while molecular mechanism validation at the cellular level remains insufficient [38–40]. To address this gap, this study employed integrated in vivo and in vitro models to comprehensively evaluate the impacts of high-glucose conditions on hepatic health, lipid metabolism, bile acid homeostasis, and mitochondrial dynamics. Furthermore, the study revealed the mechanism by which the ‌Sirt1/Pink1/Parkin axis‌ improves metabolic disorders through optimized mitochondrial quality control. These findings provide a theoretical foundation for feed formulation optimization‌ ‌and targeted metabolic intervention strategies‌, ultimately supporting the sustainable development of aquaculture‌.

## Methods

### Feeding trial and sampling

Juvenile mixed gender largemouth bass were randomly assigned to two diets: a control diet (CON) and a high-carbohydrate diet (HC) for 8-week trial starting at 8.24 ± 0.01 g initial weight. As shown in Table 1, these two diets were made, packed, and stored according to our standard laboratory procedures [41]. Briefly, all ingredients were ground into powder and mixed thoroughly by a feed mixer (A-200 T Mixer Bench Model unit, Resell Food Equipment Ltd, Ottawa, Canada). A screw-press pelletizer was used to obtain 2.0 mm pellets from the mixture containing fish oil, soy oil, and soya lecithin and water (F-26, South China University of Technology, Guangzhou, China). Pellets were dried at 16 °C in a well-ventilated condition until moisture content dropped below 10%, and then stored at −20 °C. A total of 240 fish were randomly allocated into 6 tanks (capacity: 100 L) with 30 fish per tank. There were four replicates of each diet. All fish were cultured in an experimental system with commercial feed (Tongwei Co., Ltd., China) for two weeks to adapt to the experimental conditions. During the 8-week trial, fish were fed two times per day at 9:00 and 17:00 and maintained under recirculating aquaculture system of 25–28 °C water temperature, 9.0 mg/L dissolved oxygen, 7.9–8.2 pH, and lower than 0.2 mg/L ammonia nitrogen level. After the trial, fish were anaesthetized and euthanized with MS-222 (200 mg/L; Sigma, USA) and then weighed. The viscera and liver of each fish were also weighed and photographed for the evaluation of hepatic and visceral lipid accumulation. For future analyses, tissues (including liver, heart, brain, intestine, and head kidney; *n* = 4) were promptly frozen in liquid nitrogen and stored at −80 °C.

**Table 1:** Ingredients and nutrient compositions of the experimental diets (dry matter basis)

Ingredients, %	CON	HC
Corn starch	0	20
Fish meal	45	45
Krill meal	3	3
Beer yeast	5	5
Soybean meal	10.3	10.3
Wheat gluten	10	10
Fish oil	1	1
Soy oil	1	1
Soya lecithin	1	1
Mineral premix^a^	1	1
Vitamin premix^b^	1	1
Choline chloride (50%)	0.5	0.5
Monocalcium phosphate	1	1
Vitamin C	0.2	0.2
Bone meal	20	0
Sum	100	100
Nutrient composition, %		
Crude protein	47.92	48.66
Crude lipid	6.66	6.68

^a^Mineral premix provides the following per kg of diet: MgSO_4_∙7H_2_O, 1,090 mg; KH_2_PO_4_, 932 mg; NaH_2_PO_4_∙2H_2_O, 432 mg; FeC_6_H_5_O_7_∙5H_2_O, 181 mg; ZnCl_2_, 80 mg; CuSO_4_∙5H_2_O, 63 mg; AlCl_3_∙6H_2_O, 51 mg; MnSO_4_∙H_2_O, 31 mg; KI, 28 mg; CoCl_2_∙6H_2_O, 6 mg; Na_2_SeO_3_∙H_2_O, 0.8 mg

^b^Vitamin premix provides the following per kg of diet: Vitamin B_1_, 30 mg; Vitamin B_2_, 60 mg; Vitamin B_6_, 60 mg; Nicotinic acid, 200 mg; Calcium pantothenate, 100 mg; Inositol, 100 mg; Biotin, 2.5 mg; Folic acid, 10 mg; Vitamin B_12_, 0.1 mg; Vitamin K_3_, 40 mg; Vitamin A, 10,000 IU, Vitamin E, 160 IU. Vitamin B_1_ and B_2_ are termed as thiamin and riboflavin

### Periodic acid-Schiff (PAS) staining and glycogen content detection of the liver

Liver tissue sections (1–4 µm) were fixed in 4% paraformaldehyde (Servicebio, Wuhan, China) for 24 h and embedded in paraffin. Liver glycogen staining was conducted using the periodic acid-Schiff (PAS) reaction. Images were acquired by a light NikonNi-U microscope (Nikon Corporation, Japan) with 20× magnification. The glycogen content was measured by spectrophotometry using standard commercial kits from Wuhan Abbkine Co., China (KTB1340).

### Primary hepatocyte isolation and treatment

Primary hepatocytes were isolated from juvenile healthy largemouth bass (weight: 10–20 g) livers, which feed with commercial diets (Tongwei Co., Ltd., China) two times per day at 9:00 and 17:00, and cultured following established protocols [41]. Briefly, healthy largemouth bass were first bled and sacrificed by gill cutting under sterile conditions. The livers were aseptically excised and finely minced using scissors in phosphate-buffered saline (PBS) at pH 7.4. Subsequently, the minced liver tissue underwent enzymatic digestion with trypsin (25200072; Thermo Fisher, USA) at 28 °C for 40 min. The digested liver tissue was filtered through a sterile 70 μm cell strainer to remove undissociated tissue fragments and the filtrate was collected as a single-cell suspension. Then the suspension was centrifuged at 1,000 r/min for 5 min at room temperature to collected the cell pellet for subsequent cell counting. The harvested cell pellet was cultured in low-glucose medium (66113-18508; 1,000 mg/L; Vigonob, Guangzhou, China) containing 20% FBS and 1% penicillin–streptomycin. A total of 1 × 10^6^ living cells per well were seeded into 6-well plates and culture at 28 °C in a humidified incubator with air charge of 5% CO_2_. Cells were treated with either low-glucose (LG; 1,000 mg/L) or high-glucose (HG; 4,500 mg/L) for 24, 48, and 72 h when they reached 70%–80% confluence. The cells were pretreated with various concentrations of SB203580 (p38 MAPK pathway inhibitor; S1076, Selleck Chemicals, Houston, Texas, USA) for 2 h, then treated with HG for 48 h.

### Cell viability assays

Cell viability assays were examined by Cell Counting Kit-8 (CCK8) (FD3788; Fdbio Science, Hangzhou, China). The isolated primary hepatocytes were seeded in 96-well plates (100 μL/well) at a density of 1 × 10^4^ cell/well. After 24 h, 48 h, and 72 h cultured in either LG or HG medium, 10 μL of CCK8 was added to each well 2 h prior to measuring the absorbance at 450 nm using a multifunctional microplate reader.

### Annexin V-FITC/PI staining

Cell apoptosis was evaluated with flow cytometry (BL110A; Biosharp Life Sciences, Beijing, China). Primary hepatocytes were seeded in 6-well plates and subsequently digested with EDTA-free trypsin after LG or HG treatments. According to the manufacturer's instructions, both the supernatants and cell pellets were collected and stained with 5 μL of Annexin V-FITC and 10 μL of PI staining solution, respectively. The apoptotic cells were detected by CytoFLEX flow cytometer (Beckman Coulter, Indianapolis, IN, USA).

### Assessment of mitochondrial membrane potential (MMP)

Assessment of the electrical potential across the mitochondrial membrane (ΔΨm). The MMP alterations were evaluated using the MMP assay kit containing JC-1 (C2006; Beyotime Biotechnology, Shanghai, China). Hepatocytes were initially cultured with LG and HG for 48 h, followed by washing with ice-cold PBS and staining with 1 mL of JC-1 working solution at 28 °C for 20 min. Subsequently, stained cells were examined using a Leica DM1000 fluorescence microscope (Leica Microsystems GmbH, Wetzlar, Germany).

### Measurement of ROS

Generation of intracellular ROS was identified by utilizing the H2DCF-DA probe (C-2938; Invitrogen™, Waltham, MA, USA). After LG or HG treated for 48 h, cells were incubated in serum-free DMEM with 15 μmol/L H_2_DCF-DA for 30 min, shielded from light, and then the medium were replaced with pre-warmed PBS. ROS fluorescence was visualized under a Leica SP8 STED confocal laser scanning microscope (Leica Microsystems GmbH, Wetzlar, Germany), and the intensities were measured using Image-Pro Plus software. Additionally, the level of ROS was also detected by Cytoflex flow cytometry (Beckman Coulter, Inc., Brea, CA, USA).

### Electron microscopy

For transmission electron microscopy (TEM), liver tissues and cells were fixed in 2.5% glutaraldehyde (AAPR46; Servicebio, Wuhan, China) and rinsed with PBS. The samples were dehydrated in a graded series of ethanol and embedded in pure resin overnight. In order to observe various structures within the livers and cells, a transmission electron microscope (JEM-1400 Flash, Tokyo, Japan) was used for observation and photography.

### Oil Red O staining and PAS staining

For the cellular experiments, Oil Red O staining and ‌PAS staining were performed according to the manufacturer’s protocol (cat. no. C0157 and C0142 respectively; Beyotime Institute of Biotechnology, Shanghai, China). Lipid and glycogen accumulation in cells was observed using a 20× fluorescence microscope (Leica DM1000; Leica Microsystems GmbH, Wetzlar, Germany).

### Analysis of mitochondrial-related indicators

Mitochondrial staining assay was determined by staining the cells with Mito Tracker Red (cat. no. M7521; Invitrogen; Thermo Fisher Scientific, Inc., Waltham, MA, USA) and Mito Tracker Green FM (cat. no. M7514; Invitrogen; Thermo Fisher) according to the manufacturer’s protocol. The fluorescence intensity was assessed in 1 × 10^4^ cells by flow cytometry. For mitochondrial reactive oxygen species (mROS) production and mitochondrial morphology analysis, cells were labeled with 5 μmol/L MitoTracker™ Red CM-H_2_XRos (cat. no. M7513, Thermo Fisher) for mROS and 5 μmol/L MitoTracker™ Red FM (cat. no. M22425, Thermo Fisher) in PBS at 28 °C for 30 min, followed by super-resolution imaging with a Leica SP8 STED microscope (Leica Microsystems GmbH, Wetzlar, Germany).

### Confocal microscopy

GFP-LC3 and pRK5-flag-ev were stored in our laboratory. pcDNA3.1(+)-MITO/Turbo RFP was purchased from Yunzhou Biology Company (VB211020-1156jcn). To construct the pRK5-flag-*sirt1* plasmids, *sirt1* mRNA was amplified using the following primer pairs: *sirt1* forward, 5′-ACAAGGACGACGATGACAAGATGGCGGACGGAGAGAGCAGT-3′ and reverse, 5′-AGGTCGACTCTAGAGGATCCTTAAAGGTGTGTGGTGCTCTGAG-3′; pRK5-flag-*ev* was amplified using the following primer pairs: pRK5-flag-*ev* forward, 5′-GGATCCTCTAGAGTCGACCTGCAG′ and reverse, 5′-CTTGTCATCGTCGTCCTTGTAGTCCA-3′. Subsequently, Sirt1 and pRK5-flag-*ev* were ligated by homologous recombination (cat. no. C5891; Clone Smarter Technologies, Beijing, China), and identified using EcoRI enzyme digestion. siRNA targeting Sirt1 (5′-GCCAAUGAGGCCACAUCAATT-3′), and the negative control (5′-UUGAUGUGGCCUCAUUGGCTT-3′) were synthesized by Shanghai Sangon Biotech Co., Ltd. Plasmids and si-Sirt1 were transfected into the cells with jetPRIME^®^ (101000046; Polypolus, Strasbourg, France) in a laser confocal culture dish (80100215). To stain the acidic compartments, live cells were stained with 50 nmol/L LysoTracker Red (L7528; Thermo Fisher) for 2 h at 28 °C in the dark and fixed with 4% paraformaldehyde (AAPR12). Subsequent, cells were permeabilized using 0.1% Triton X-100 (AAPR96) and blocked using 2% BSA (AAPR305) in TBST. The primary antibody dilutions were as the follows: Tom20 (1:100), Sirt1 (1:100), Flag (1:100), Pink1 (1:100), p-P38 (1:100). The membranes were washed with TBST and incubated with appropriate secondary antibodies (Alexa Fluor goat anti-rabbit 594, 1:100) for 1 h. Finally, ProLong Gold Antifade (P36941) was used to stain the nuclei and prevent fluorescence quenching. The slips were imaged using a Leica SP8 STED confocal laser scanning microscope (Leica Microsystems GmbH).

### Quantitative real-time PCR (RT-PCR) and western blot

Total RNA was extracted from various tissues of largemouth bass and cells using RNAiso Plus reagent (9109, Takara Bio, Inc., Japan) and reverse transcribed to cDNA on the specification (AK2601; Takara Bio, Inc., Japan). RT-PCR was performed using the Roche Light Cycler 480II Real-Time System (Switzerland), and the gene expression levels were normalized with reference to *ef-1α* (GenBank accession no. KT827794) using the 2^−ΔΔCq^ method. PCR amplification primer sequences were shown in Table 2.

**Table 2:** Sequences of primers used in this study

Genes	Forward primers (5′→3′)	Reverse primers (5′→3′)	Sources/GenBank No.
*cpt1*	TTCCCCTTTATTGACTTTGGC	AGAACTTCCCTTTGTCCCTGTAA	[42]
*hsl*	AGGACAGGACAGTGAAGAGTTGC	CAGATAATTCTCATGGGATTTGG	NW_024040152.1
*atgl*	CCATGATGCTCCCCTACACT	GGCAGATACACTTCGGGAAA	NW_024044570.1
*ppar-α*	CCACCGCAATGGTCGATATG	TGCTGTTGATGGACTGGGAAA	NW_024041484.1
*fas*	CAGCCCTTGACTCATTCCG	CGCAGACTACGACCCGACAG	NW_024041262.1
*acc1*	ATCCCTCTTTGCCACTGTTG	GAGGTGATGTTGCTCGCATA	NW_024044681.1
*srebp1*	AGTCTGAGCTACAGCGACAAGG	TCATCACCAACAGGAGGTCACA	NW_024040041.1
*ppar-γ*	CCTGTGAGGGCTGTAAGGGTTT	TTGTTGCGGGACTTCTTGTGA	NW_024043372.1
*lpl*	ACCAGCACTACCCGACCTCC	CAGACTGTAACCCAGCAGATGAAT	NW_024040374.1
*apob*	AGGCTGGGTGTTGTTGATGG	GAGAGCTGAGGGATGTTCTTGTTTAG	NW_024041039.1
*apob100*	CAACTTGAAAATGTCCCTCTCTC	TCTTAATGACTGATGACTCTGCCT	[42]
*fabp1*	GAACCTCAAGGAGAGCCAGAA	CACCGTCCACCGAGATAATAGT	NW_024044459.1
*cyp7a1*	CTGGGCTTCACAGGCTAACACC	TTCAGTGTGGGGTCGTTGGG	[42]
*cyp8b1*	TAGACAGCGGCAACCAGGAG	CCGTGCTTTTGTTTCATCCTATC	[42]
*fxr*	TTGAGCCGAAAGATGCCCAA	CCGATCTGGTGTCAGGATGG	NW_024044570.1
*rxrα*	GTCTGTCCAACCCTGGTGAG	TCGCCGATGAGCTTGAAGAA	NW_024041151.1
*shp*	AACCAACTCTTGCTGAAGTCCAC	TTCAACAAACGACAAGGCACTC	[42]
*hmgcr*	GGTGGAGTGCTTAGTAATCGG	ACGCAGGGAAGAAAGTCAT	NW_024044459.1
*fgfr4*	ATTCAATCGGATTCGCTCACCAGTC	AGGAAACCACAGGCATAGATGATGATG	XM_038708053.1
*fgf19*	AGGCTGTGTTGTCATCAAAGGAGTAG	GTCTCTGCTGTAGGTGTGCGATG	XM_038702190.1
*fgf21*	ATCTCGCTGACTCCAACCCTCTC	TTACTTCCCGATACTCTCCCATCCAG	XM_038736351.1
*cytb*	CTGCCGCCACAGTAATCCATCTAC	CGAACCCGAGCAAGTCTTTATAGGAG	NC_008106.1
*nrf2*	CTCTGTTCCCAGTATGGCCC	GAAGGGAGGCTTGTTTGGGA	NW_024040596.1
*keap1a*	AAACGTCCCACACGTGACTC	ACACAACATCTCCTGCCGTC	NW_024041039.1
*keap1b*	CCTGTGTGATCAGTGGGCTT	ATGTGATCCACCAACCGCAT	NW_024040263.1
*nqo1*	GACATCATCGGCGACCTGAA	GCAGGAACGCTGAACCAGTA	NW_024044348.1
*ennp1*	GCAGTGATGGAAACGGAGGGAAG	CCAAGGACACCAGGATGAGAGGAG	XM_038726340.1
*gsy2*	CCCTGATCGCCTGGTTCTTCAAAG	CAGCCTGCCACTCGTGGAAATG	XM_038738888.1
*acadm*	TGGCTGAGATGGCAATGAAGGTG	TTGGCGATGGAGGCGTAGTAGG	XM_038717481.1
*bcl-2*	TGCCTTTGTGGAGCTGTATG	GGAAGAGGAGGAGGAGGATG	[42]
*bax*	TCTTCACTCAGTCCCACAAA	ATACCCTCCCAGCCACC	XM_038704178.1
*bad*	CACATTTCGGATGCCACTAT	TTCTGCTCTTCTGCGATTGA	XM_038730645.1
*p53*	AGATTGAATGGTGGTGGG	GTTCTGGCGGACTGGA	[42]
*casp3*	GCTTCATTCGTCTGTGTTC	CGAAAAAGTGATGTGAGGTA	[42]
*casp8*	GAGACAGACAGCAGACAACCA	TTCCATTTCAGCAAACACATC	[42]
*casp9*	CTGGAATGCCTTCAGGAGACGGG	GGGAGGGGCAAGACAACAGGGTG	[42]
*casp10*	CAAACCACTCACAGCGTCTACAT	TGGTTGGTTGAGGACAGAGAGGG	[42]
*sirt1*	GCCAGCGGAGAAGGAAGCAAAG	CCAGCAGTCCCAGTCCATTGTTG	XM_0387368121
*sirt2*	AGGCTACAGGAGACACAGAGATGG	TAGCGTGCCACTCCGTCCAG	XM_038693630.1
*sirt3*	GATCCCTCCTGCGATGTTGGTTG	CTCGTCTCCCTCGTACCTCTTCAG	XM_038701377.1
*sirt4*	CGGATGCGGTGCTGGTTGTG	CTCGTGGACCCAATGTTCAGGATG	XM_038696269.1
*sirt5*	ATGGCTGTCACGGTCTCCTGAG	ACCACGAGGCAGAGGTCACAG	XM_038715062.1
*sirt6*	ACCTCTGAGTCCACCACGATGTC	GCCGAAGCCGTCTCCTCTTTAAC	XM_038694835.1
*sirt7*	GCCGCTGTGTTGTTGTTGCATAG	ACCTCCTCCTGCTTCCTCTTGAG	XM_038714134.1
*ef-1α*	TGCTGCTGGTGTTGGTGAGTT	TTCTGGCTGTAAGGGGGCTC	KT827794.1

A mixture of protease inhibitors (FD1002; Fdbio science, Hangzhou, China) was added to RIPA lysis buffer (FD009; Fdbio science, Hangzhou, China; 1:100) to extract total protein from livers and cells. All details of the primary antibodies and corresponding secondary antibodies used were stated in Table 3. Bands were visualized using an Azure 300 ultra-sensitive chemiluminescence imager (Azure Biosystems, USA). Protein levels were standardized to β-actin levels and quantified using Image-Pro Plus software.

**Table 3:** Antibodies used in this study

Antibody	Dilution ratio	Supplier information
ACC	1:1,000	Cell Signaling Technology, #3662
Phospho-ACC(Ser69)	1:1,000	Cell Signaling Technology, #3661
AMPKα	1:1,000	Cell Signaling Technology, #5831
Phospho-AMPKα (Thr172)	1:1,000	Cell Signaling Technology, #2535
JNK1	1:1,000	Cell Signaling Technology, #3708
Phospho-SAPK/JNK (Thr183/Tyr185)	1:1,000	Cell Signaling Technology, #4668
p38MAPK	1:10,000	Cell Signaling Technology, #9212
Phospho-p38 MAPK (Thr180/Tyr182)	1:10,000	Cell Signaling Technology, #4511
p44/42 MAPK	1:1,000	Cell Signaling Technology, #4695
Phospho-p44/42 MAPK (Thr202/Tyr204)	1:1,000	Cell Signaling Technology, #4370
Hsp60	1:1,000	Proteintech, #15282
Drp1	1:1,000	Cell Signaling Technology, #43110 T
Tom20	1:1,000	Cell Signaling Technology, #43110 T
LC3B	1:1,000	Sigma, # L7543
PARK2/Parkin	1:1,000	Proteintech, #66674
Pink1	1:1,000	Cell Signaling Technology, #14060 T
Sirt1	1:1,000	Cell Signaling Technology, #9475
DYDDDDK	1:1,000	Proteintech, #20543/66008
DyLight™ 488 Goat anti-Rabbit IgG	1:100	Invitrogen, #35532
DyLight™ 488 Goat anti-Mouse IgG	1:100	Invitrogen, #35502
Alexa Fluor™ Plus 647 Rabbit IgG (H + L)	1:100	Invitrogen, #A32733TR
DyLight™ 594 Goat anti-Rabbit IgG	1:100	Invitrogen, #35561
Goat Anti-Rabbit IgG	1:10,000	Proteintech, #SA00001-2
Goat Anti-Mouse IgG	1:10,000	Proteintech, #SA00001-1

### Statistical analysis

Analysis of variance (ANOVA) or unpaired/paired *t*-tests of three independent repeats were performed with GraphPad Prism 8 (GraphPad Software, Inc., La Jolla, CA, USA). Data from three or four independent biological replicates were expressed as mean ± SEM. Normality was assessed by Shapiro-Wilk test, and homogeneity of variance was confirmed by Brown-Forsythe test. For comparisons between multiple groups, one-way ANOVA with Tukey’s post hoc test was applied when parametric assumptions were met; otherwise, Kruskal–Wallis test with Dunn’s correction was used. Paired/unpaired *t*-tests (two-tailed) were selected for two-group comparisons after verifying Gaussian distribution.

## Results

### High-glucose treatment induced hepatic glycolipid accumulation, liver damage and oxidative stress in both largemouth bass and primary hepatocytes

**Fig. 1:**
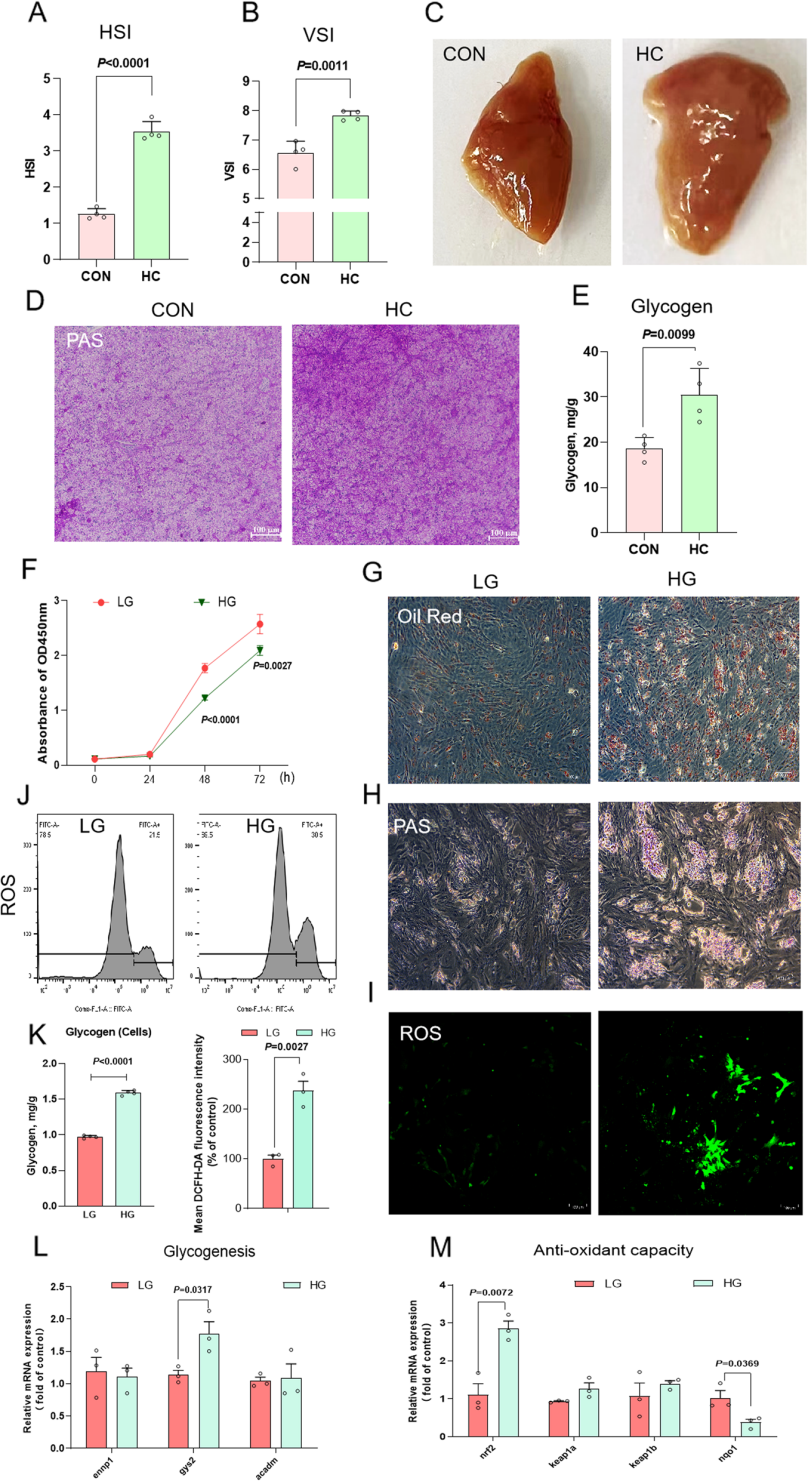
High-glucose treatment induced hepatic glycolipid accumulation, liver damage and oxidative stress in both largemouth bass and primary hepatocytes. **A** Hepatosomatic index (HSI, *n* = 4). **B** Viscerosomatic index (VSI, *n* = 4). **C** Liver morphology. **D** PAS staining of liver sections. **E** The content of glycogen in the liver. **F** Cell Counting Kit-8 test (*n* = 3). **G** Oil Red O staining of cells. **H** PAS staining of cells. **I** Intracellular ROS were determined using a fluorescence microscope. **J** The measurement of intracellular ROS. **K** The content of glycogen in cells (*n* = 4). **L** Relative expression of glycogenesis-related genes (*ennp1*, *gys2*, and *acadm*) in cells (*n* = 3). **M** Relative expression of anti-oxidant capacity-related genes (*nrf2*, *keap1a*, *keap1b* and *nqo1*) in cells (*n* = 3)

Following the 8-week experimental period, compared to the CON diet, the hepatosomatic index (HSI) and viscerosomatic index (VSI) were significantly increased in the largemouth bass fed HC diet (Fig. 1A and B). Consistent with the HSI and VSI, HC-fed largemouth bass showed obvious white liver and enlargement (Fig. 1C). PAS staining in the liver tissue of largemouth bass further corroborated that HC diet caused profound glycogen accumulation (Fig. 1D). Correspondingly, hepatic glycogen content in the HC diet was elevated by approximately 50% relative to the CON diet (Fig. 1E). Largemouth bass primary hepatocytes were exposed to a high-glucose medium to replicate in vivo conditions associated with high-carbohydrate intake, aiming to elucidate the impact of a high-carbohydrate diet on hepatic glycolipid accumulation. The results indicated that HG treatment significantly inhibited cell proliferation at 48 and 72 h (Fig. 1F). Consequently, a 48-h treatment duration was chosen for subsequent high-glucose exposure in primary hepatocytes. The results of Oil Red O staining and PAS staining proved that HG treatment promoted the accumulation of lipid droplets and glycogen in primary hepatocytes (Fig. 1G and H). Meanwhile, we also evaluated the expression of glycogenesis-related genes and the content of glycogen in primary hepatocytes. The results exhibited that HG treatment induced glycogen synthesis, as evidenced by an increase in the mRNA levels of *gsy2* (Fig. 1L) and the content of glycogen (Fig. 1K). Moreover, generation of ROS was observed by fluorescence microscopy to detect whether HG treatment can result in oxidative stress. In primary hepatocytes, HG treatment increased mean fluorescence intensity (MFI) of H_2_DCF-DA dye, indicating elevated ROS production as compared to LG treatment (Fig. 1I). Flow cytometry analysis also demonstrated that HG treatment resulted in approximately 9% increased intracellular ROS levels in primary hepatocytes (Fig. 1J). Additionally, the gene expression level of *nrf2* was up-regulated, and the gene expression levels of *nqo1* was down-regulated after HG treatment (Fig. 1M).

### High-glucose treatment disturbs fatty acid metabolism, bile acid metabolism and activates MAPK signal pathway in both largemouth bass and primary hepatocytes

**Fig. 2:**
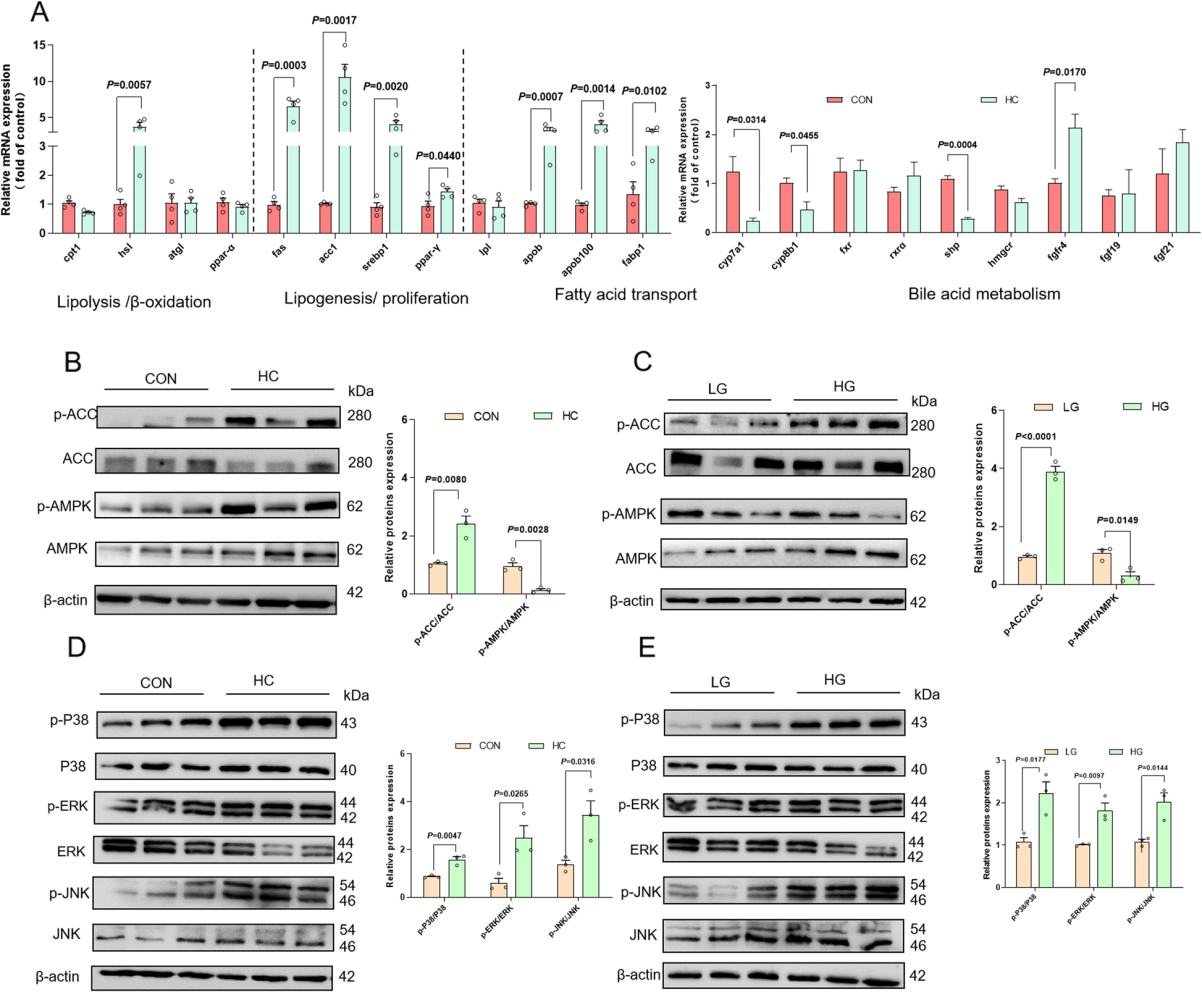
High-glucose treatment disturbs fatty acid metabolism, bile acid metabolism and activates MAPK signal pathway in both largemouth bass and primary hepatocytes. **A** Relative expression of lipolysis/β-oxidation-related genes (*cpt1*, *hsl*, *atgl* and *ppar-α*), lipogenesis/proliferation-related genes (*fas*, *acc1*, *srebp1* and *ppar-γ*), fatty acid transport -related genes (*lpl*, *apob*, *apob100* and *fabp1*) and bile acid metabolism-related genes (*cyp7a1*, *cyp8b1*, *fxr*, *rxrα*, *shp*, *hgmcr*, *fgfr4*, *fgf19*, and *fgf21*) in the livers of largemouth bass (*n* = 4). **B** Relative expression of fatty metabolism-related proteins (p-ACC, ACC, p-AMPK, and AMPK) in the livers of largemouth bass (*n* = 3). **C** Relative expression of fatty metabolism-related proteins (p-ACC, ACC, p-AMPK, and AMPK) in cells (*n* = 3). **D** Relative expression of MAPK signal pathway (p-P38, P38, p-ERK, ERK, p-JNK, and JNK) in the livers of largemouth bass (*n* = 3). **E** Relative expression of MAPK signal pathway (p-P38, P38, p-ERK, ERK, p-JNK, and JNK) in cells (*n* = 3)

Given that HC diet can lead to fat accumulation in visceral tissues, we further investigated the impact of HC diet on fatty acid metabolism and bile acid metabolism. Compared with CON diet, HC diet resulted in the upregulation of lipolysis/fatty acid oxidation, lipogenesis and fatty acid transport-associated genes, as evidenced by upregulated expression of *atgl*, *fas*, *acc1*, *srebp1*, *ppar-γ*, *apob*, *apob100* and *fabp1*. The gene expression levels of *cyp7a1*, *cyp8b1*, and *shp* were down-regulated, and the gene expression level of *fgfr4* was up-regulated in largemouth bass fed HC diet (Fig. 2A). Western blot results indicated that HC diet effectively inhibited the phosphorylation of AMPK and promoted the phosphorylation of ACC (Fig. 2B). To further characterize the impact of high-glucose treatment on fatty acid metabolism, the phosphorylation of in vivo-validated AMPK and ACC were further analyzed in an in vitro model. Consistent with the in vivo results, HG treatment activated the phosphorylation of ACC and suppressed the phosphorylation of AMPK (Fig. 2C). Since Fgfr4 activation has been shown to initiate receptor tyrosine kinase signaling cascades, thereby activating the c-JUN and ERK signaling pathways [43], we further investigated whether HC diet might activate the MAPK signal pathways. Compared with CON diet, HC diet increased the phosphorylation of ERK, JNK and p38MAPK (Fig. 2D). As indicated in Fig. 2E, the phosphorylation of ERK, JNK and p38MAPK were up-regulated after HG treatment. Thus, both in vivo and in vitro experiments demonstrated that high-glucose treatment could promote fat synthesis by regulating the AMPK/ACC/SREBP-1 pathway and activate ERK, JNK and p38MAPK signal pathway.

### High-glucose treatment damages mitochondria function in both largemouth bass and primary hepatocytes

**Fig. 3:**
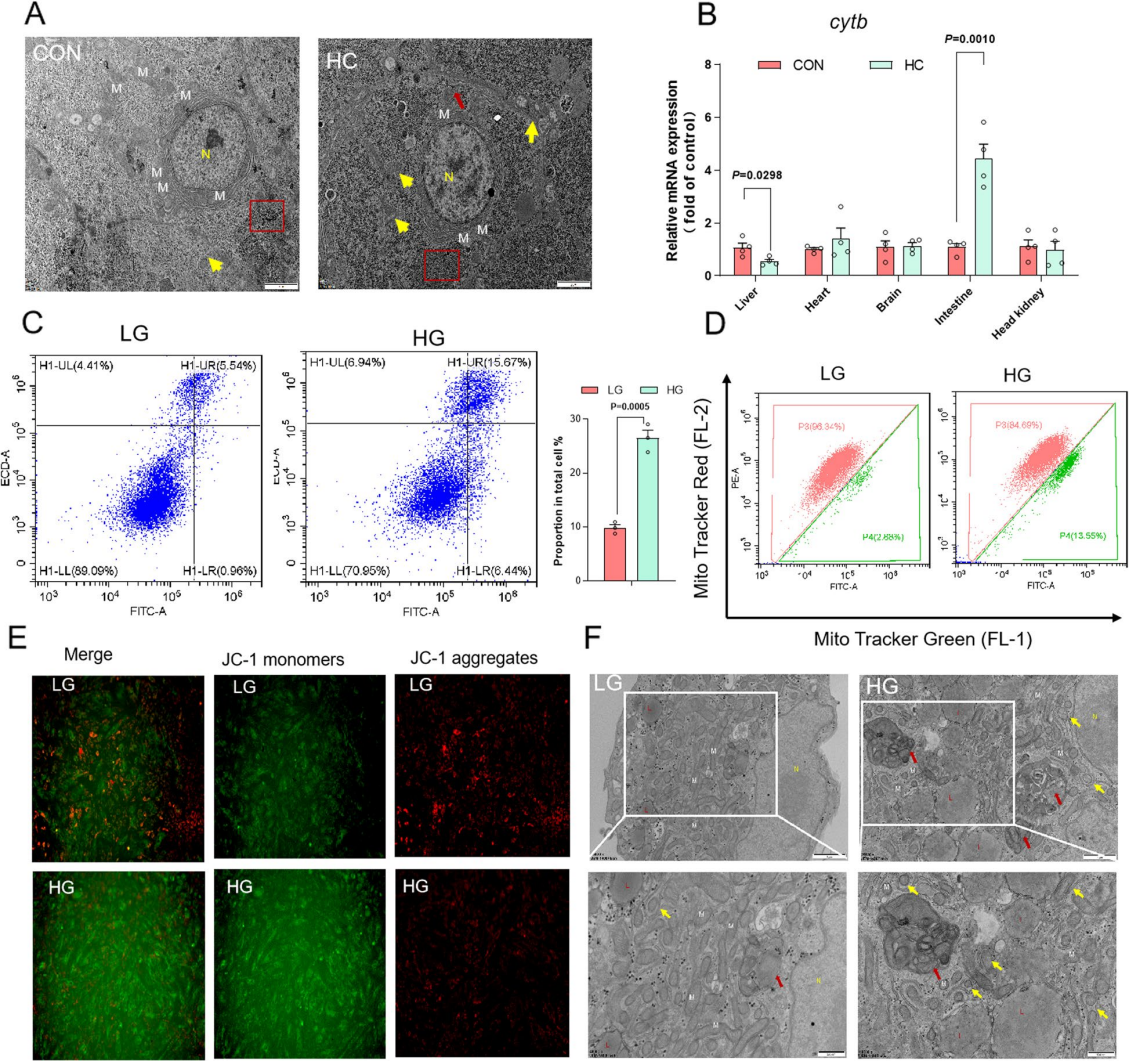
High-glucose treatment damages mitochondria function in both largemouth bass and primary hepatocytes. **A** The ultramicroscopic characteristics and structure of livers under electron microscopy. N: Nucleus; Red frame: Glycogen; Yellow arrow: Damaged mitochondria; M: Normal mitochondria; Scale bar, 2 μm. **B** Relative expression of *cytb* genes in different tissue of largemouth bass fed two different diets (*n* = 4). **C** Flow cytometry for apoptosis (*n* = 3). **D** Mito Tracker Red and Mito Tracker Green staining were measured using flow cytometry in cells (*n* = 3). **E** Mitochondrial membrane potential (MMP) analyzed by fluorescence microscope. **F** The ultramicroscopic characteristics and structure of cells under electron microscopy. N: Nucleus; L: Lipid droplet; M: Normal mitochondria; Red frame: Glycogen; Red arrow: Damaged mitochondria. Scale bar, 1 μm

Transmission electron microscopy (TEM) was used to further visualize the livers' ultramicroscopic characteristics and structural attributes. Largemouth bass fed HC diet exhibited a reduced mitochondria number, an increased damaged mitochondria and a large glycogen accumulation (Fig. 3A). It was well-established that mitochondrial *cytb* transcription decreases with mitochondrial mass [44]. HC diet markedly decreased the expression of *cytb* in liver and elevated the expression of *cytb* in intestine, whereas the expression of the other tissues did not significantly change (Fig. 3B). Other than that, flow cytometry analysis revealed that HG treatment promoted cell apoptosis (Fig. 3C). Mito Tracker Red/Mito Tracker Green intensity ratio was significantly decreased in HG-treated cells, indicating an increase in the proportion of damaged mitochondria (Fig. 3D). The decline in mitochondrial membrane potential serves as an initial indicator of cell apoptosis. HG treatment resulted in a significant increase in the number of cells exhibiting depolarized mitochondria (green), indicating a decline in mitochondrial membrane potential in primary hepatocytes (Fig. 3E). To visualize the morphological changes in primary hepatocytes treated HG, the cellular ultramicroscopic structures were also view by TEM. The mitochondria of HG-treated cells exhibited significant damage, characterized by a decrease in the number of mitochondria, mitochondrial swelling with partial vacuolation (yellow arrow), an increase in the number of lipid droplets and an increase in the number of mitophagosomes (red arrow) (Fig. 3F). This was consistent with what we observed in vivo.

### High-glucose treatment affects mitochondrial morphology and mitochondrial dynamics in primary hepatocytes

**Fig. 4:**
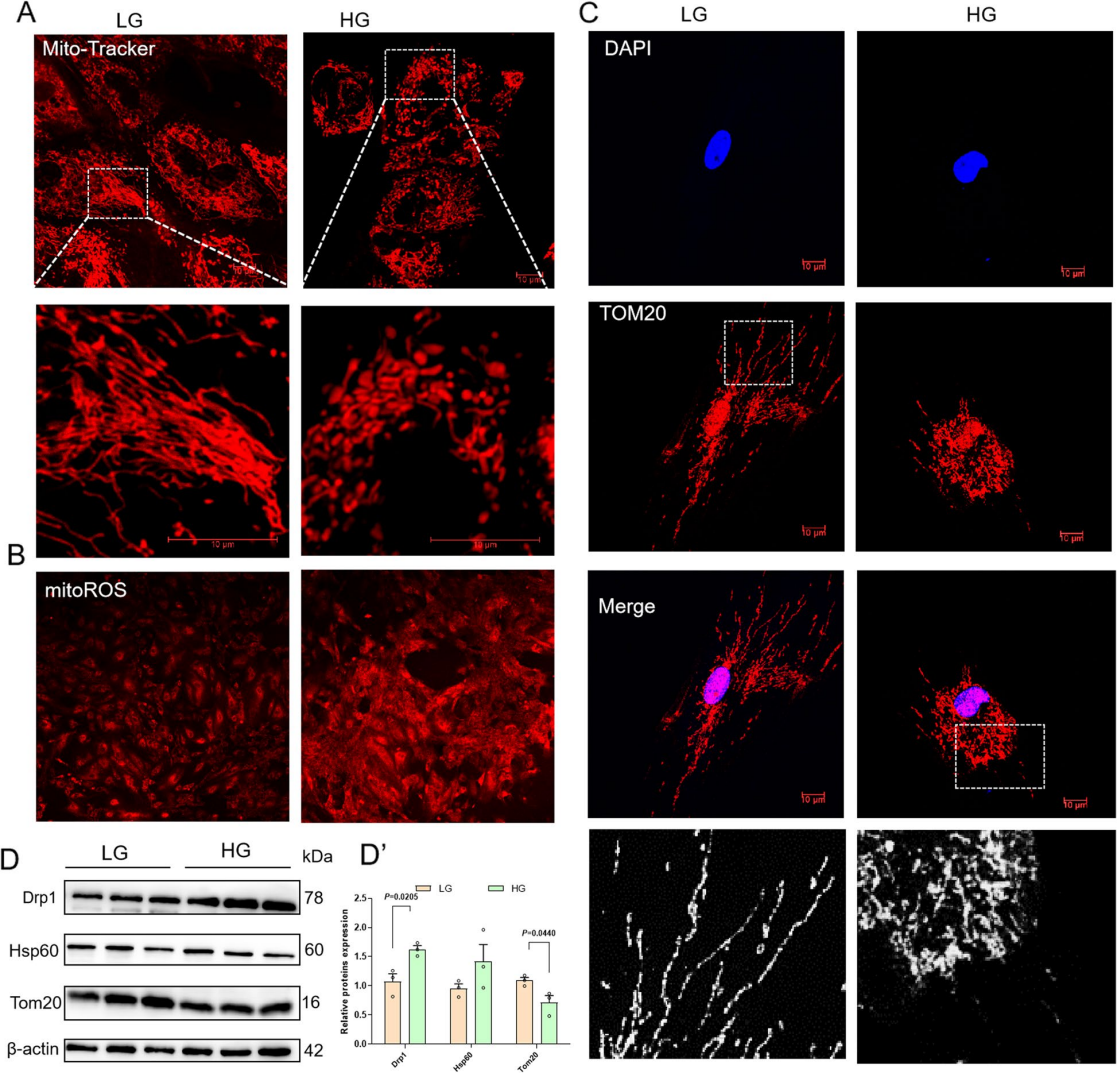
High-glucose treatment affects mitochondrial morphology and mitochondrial dynamics in primary hepatocytes. **A** Morphological changes in the mitochondria of cells. Scale bar, 10 μm. **B** Intensity of mROS in cells. **C** Fluorescence photomicrograph of Tom20 examined in cells. Scale bar, 10 μm. **D** The expression of mitochondrial dynamics-related proteins (Tom20, Hsp60, and Drp1) in cells (*n* = 3)

In the in vitro experiments, mitochondrial fusion and fission were initially detected using Mito-Tracker Red staining, and morphological alterations were observed via confocal microscopy. Under normal physiological conditions, mitochondria typically exhibit large, elongated structures with distinct networks. However, our results revealed that a significant reduction in mitochondrial length after HG treatment, indicating that HG treatment facilitated mitochondrial fission and degradation (Fig. 4A). Furthermore, the results showed that HG treatment notably elevated mitochondrial reactive oxygen species (mitoROS) production (Fig. 4B). A mitochondrial marker, Tom20, was immunofluorescence stained to further assess mitochondrial morphology. The results indicated that mitochondrial length decreased and division increased after HG treatment, which further indicated that HG treatment would lead to mitochondrial fracture and promote mitochondrial fission (Fig. 4C). In addition, the expression of related proteins was determined by western blot, which indicated a decrease in Tom20 and an increase in Drp1 levels following HG treatment, suggesting that HG treatment may play an important role in mitochondrial fission (Fig. 4D).

### High-glucose treatment promotes mitophagy and autophagy flow in primary hepatocytes

**Fig. 5:**
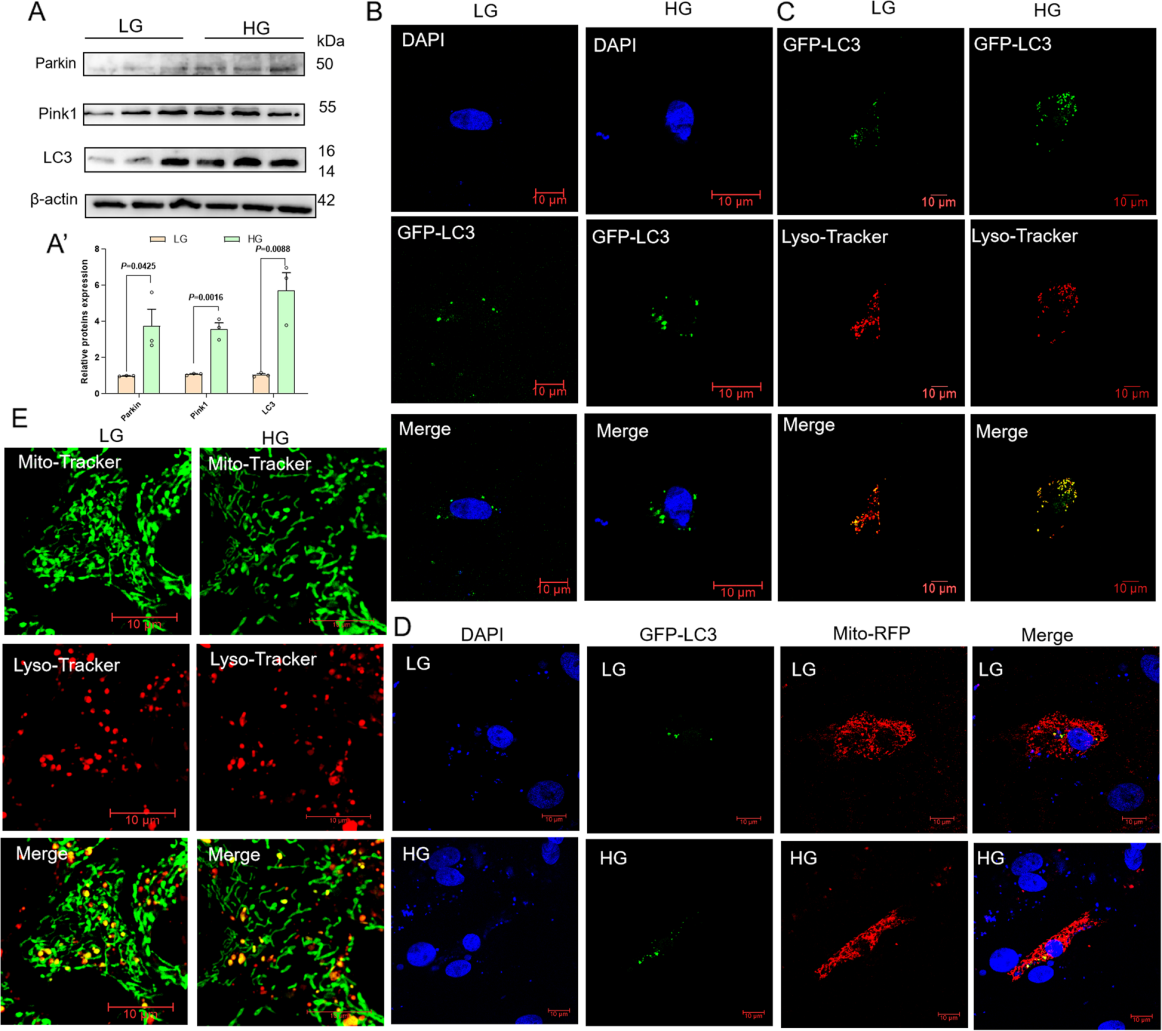
High-glucose treatment promotes mitophagy and autophagy flow in primary hepatocytes.** A** Relative expression of mitophagy-related proteins (LC3II, Pink1 and Parkin) in cells (*n* = 3). **B** GFP-LC3 staining was used to assess autophagy. Scale bar, 10 μm. **C** The cells were stained using Lyso-Tracker Red (50 μmol/L; red) and transfected with GFP-LC3 (green) to detect colocalization of autophagosomes and lysosomes. Scale bar, 10 μm. **D** Mito-RFP (red) and GFP-LC3 (green) were co-transfected to detect the binding of autophagosomes to mitochondria. Scale bar, 10 μm. **E** The cells were stained using Lyso-Tracker Red (50 μmol/L; red) and Mito-Tracker Green (50 nmol/L; green) to detect the binding of lysosomes to mitochondria. Scale bar, 10 μm

Mitochondrial dynamics, encompassing fusion and fission processes, alongside autophagy, function as critical quality control mechanisms for maintaining mitochondrial homeostasis. An array of proteins associated with mitophagy, notably Pink1 and Parkin, in addition to autophagic proteins such as LC3II, were investigated (Fig. 5A). In response to HG treatment, there was a marked upregulation of Pink1, Parkin, and LC3II, indicating that HG treatment may induce mitochondrial damage linked to Pink1/Parkin-mediated mitophagy. In order to further investigate the regulatory mechanism of HG treatment on autophagy, primary hepatocytes were transfected with a GFP-LC3 plasmid to quantify autophagosome formation. The results demonstrated that LG treated cells exhibited a limited number of autophagosomes, whereas a substantial increase in autophagosome formation was observed following HG treatment (Fig. 5B). Subsequently, laser confocal microscopy was employed to assess the colocalization of autophagosomes with lysosomes, mitochondria with lysosomes, and autophagosomes with mitochondria. The results demonstrated that HG treatment significantly enhanced GFP-LC3 lysosomal fusion (Fig. 5C), indicating increased autophagic flux. Furthermore, HG treatment promoted GFP-LC3 and Mito-RFP colocalization (Fig. 5D), while simultaneously inducing distinct colocalization between Mito-Tracker Green-labeled mitochondria and Lyso-Tracker Red-labeled lysosomes (Fig. 5E). Collectively, these findings confirm that HG treatment activates Pink1/Parkin-mediated mitophagy through the specified pathway in vitro.

### High-glucose treatment causes mitochondrial apoptosis in primary hepatocytes

**Fig. 6:**
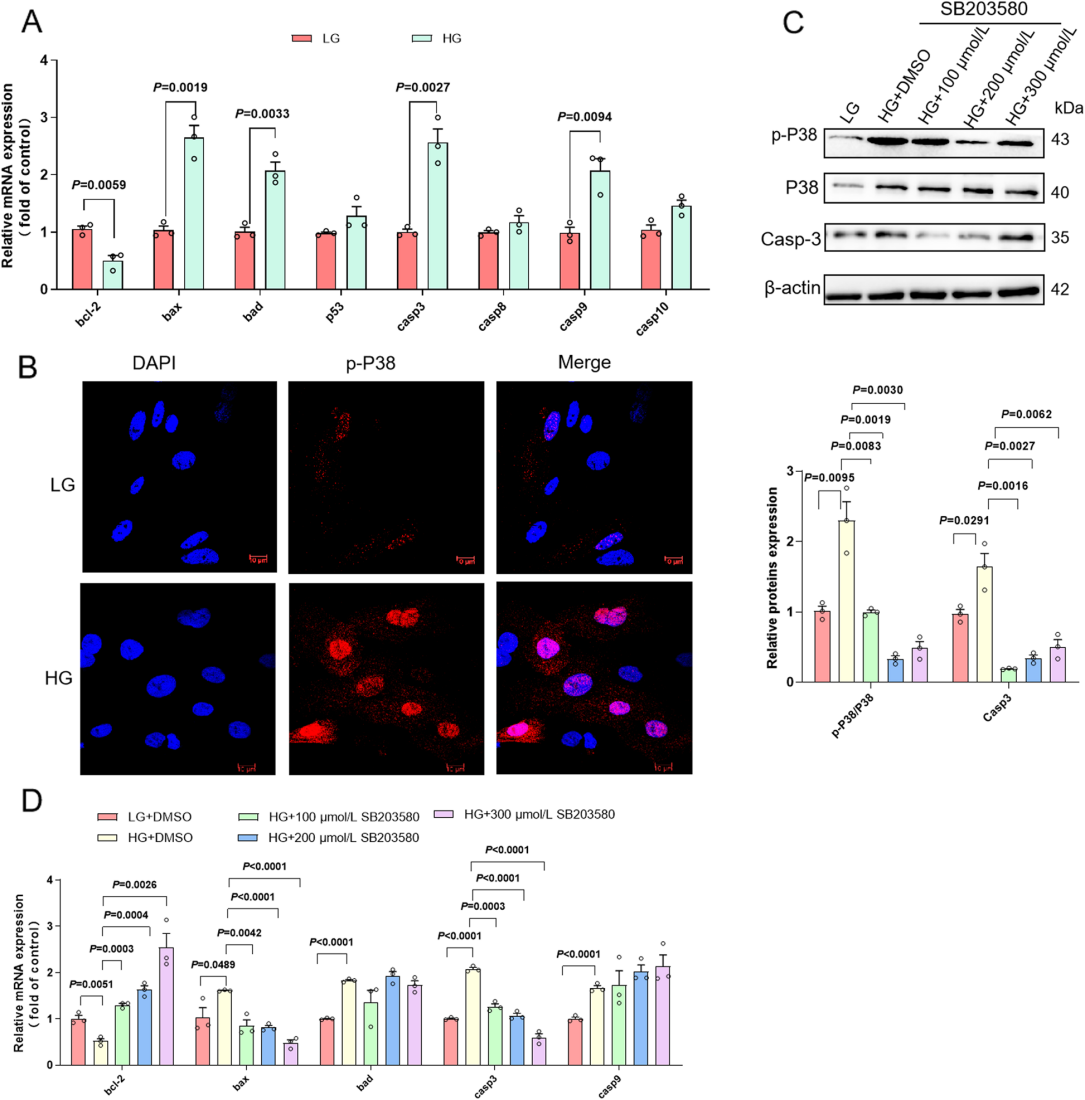
High-glucose treatment causes mitochondrial apoptosis in primary hepatocytes. **A** The expression levels of *bcl-2*, *bax, bad*, *casp3* and *casp9* of primary hepatocytes (*n* = 3). **B** immunofluorescence for p-P38. **C** Primary hepatocytes were pretreated with SB203580, expression levels of p-P38, P38 and Casp3 were analyzed using western blotting (*n* = 3). **D** Primary hepatocytes were pretreated with SB203580, expression levels of *bcl-2*, *bax, bad*, *casp3* and *casp9* were analyzed using RT-PCR (*n* = 3)

In order to elucidate the impact of high-glucose treatment on mitochondrial apoptosis. RT-PCR was used to quantify the expression of Bcl-2 family (*bcl-2*, *bax* and *bad*) and Caspase family (*casp3*, *casp8*, *casp9*, *casp10* and *p53*). As expected, HG treatment boosted mitochondrial apoptosis, as evidenced by elevated expressions of *bax*, *bad*, *casp3*, and *casp9*, and decreased expressions of *bcl-2* (Fig. 6A). P-P38 immunofluorescence was markedly enhanced both in the cytoplasm and nucleus in HG treated primary hepatocytes (Fig. 6B). Thus, we ensured that HG treatment significantly promoted the p38MAPK signal pathway. Primary hepatocytes were pretreated with different concentrations of SB203580 for 2 h and incubated with HG for another 48 h. The gene and protein levels of Casp3 were significantly downregulated by the addition of SB203580 (Fig. 6C and D). Furthermore, the gene expression levels of *bcl-2*, *bax *and *casp3* were significantly altered in cells treated with HG in the presence of SB203580 (Fig. 6D). The results suggested that high-glucose treatment can induce mitochondrial apoptosis by activating the p38MAPK/bcl-2/Casp3 signaling pathway.

### Sirt1 suppresses high-glucose-induced Pink1/Parkin-mediated mitophagy

**Fig. 7:**
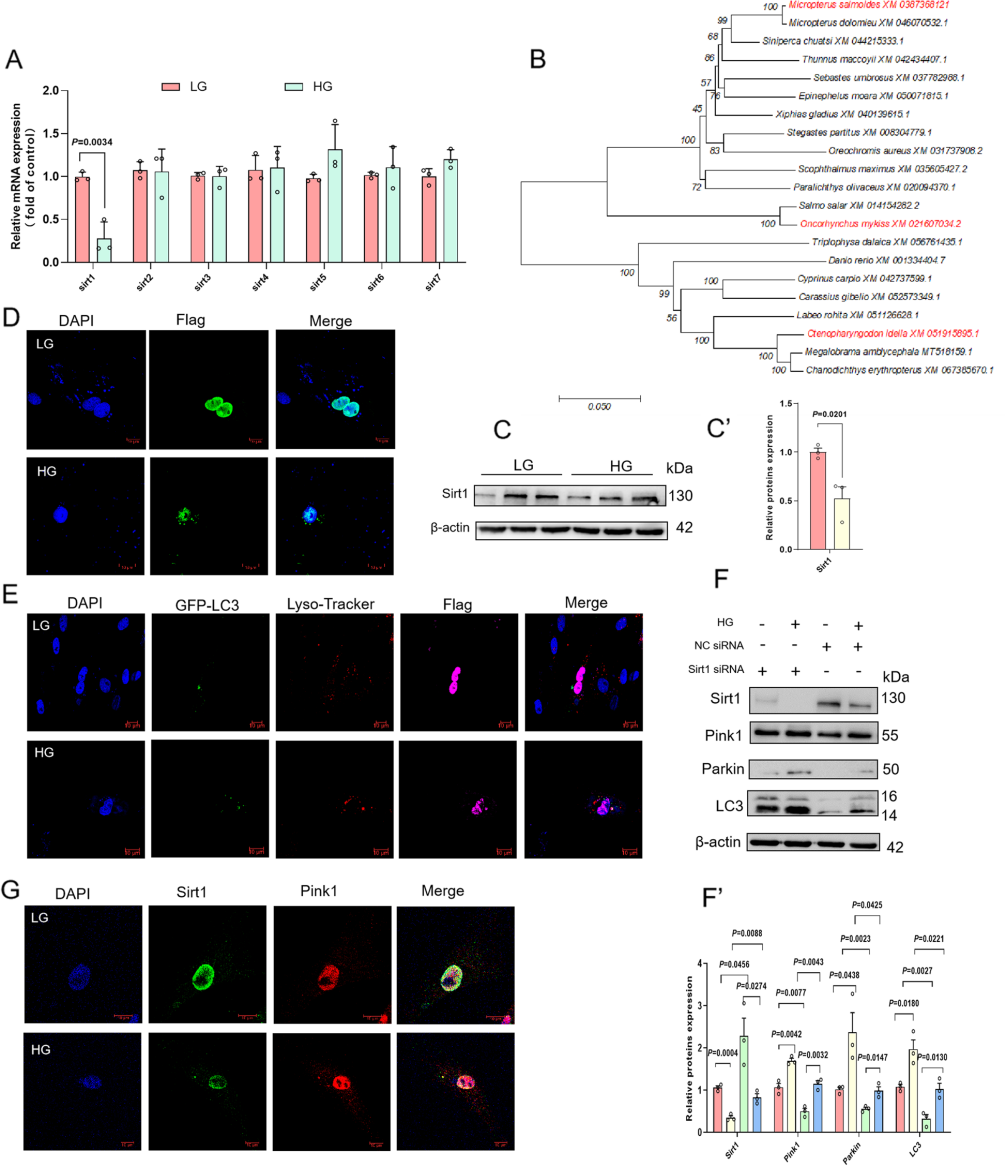
Sirt1 suppresses high-glucose-induced Pink1/Parkin-mediated mitophagy. **A** Relative expression of Sirtuins family genes (*sirt1*-*sirt7*) in cells (*n* = 3). **B** The phylogenetic tree of Sirt1 protein base sequence was constructed by MEGA 3.1 software. **C** Relative expression of Sirt1 protein in cells (*n* = 3). **D** The distribution of exogenous Sirt1 was detected by immunofluorescence. Scale bar, 10 μm. **E** The cells were co-transfected with GFP-LC3 (green) and Flag-Sirt1 (purple), and stained with Lyso Tracker Red (red) to visualize the binding of autophagosomes, lysosomes, and the Sirt1 protein. Scale bar, 10 μm. **F** Effects of si-Sirt1 transfection on the protein expression levels of LC3II, Pink1, Parkin and Sirt1 (*n* = 3). **G** The binding of endogenous Sirt1 to Pink1 protein was detected by confocal laser. Scale bar, 10 μm

Sirtuins‌ are a class of NAD^+^-dependent deacetylases that regulate metabolic processes [45]‌. In the Sirtuins family (*sirt1*–*sirt7*), HG treatment specifically downregulated *sirt1*‌ expression without affecting *sirt2*–*sirt7* (Fig. 7A)‌. Phylogenetic analysis revealed that the NAD^+^-binding domain‌ and ‌catalytic core sequence‌ of Sirt1 are highly conserved across species, underscoring its evolutionary stability in energy metabolism regulation‌. Notably, the *sirt1* gene of *Micropterus salmoides* exhibited closer phylogenetic proximity to carnivorous fish such as *Micropterus dolomieu* and *Siniperca chuatsi*, while forming a distinct evolutionary clade from *Oncorhynchus mykiss* and *Ctenopharyngodon idella* (Fig. 7B), suggesting a potential link between Sirt1-mediated metabolic regulation and dietary/ecological adaptations‌. Western blot‌ confirmed that HG treatment significantly reduced Sirt1 protein levels (Fig. 7C)‌. ‌Immunofluorescence staining‌ of exogenous Sirt1 revealed its predominant nuclear localization in primary hepatocytes under normal conditions, whereas HG treatment induced cytoplasmic Sirt1 puncta formation (Fig. 7D)‌. ‌Confocal microscopy‌ further demonstrated nuclear-to-cytoplasmic translocation of Sirt1 under high-glucose treatment, with cytoplasmic Sirt1 colocalizing with ‌autophagosomes‌ (GFP-LC3) and ‌lysosomes‌ (LysoTracker), implicating its role in modulating the autophagy-lysosome pathway to counteract metabolic stress (Fig. 7E)‌. ‌SiRNA-mediated Sirt1 knockdown‌ in HG treatment cells markedly upregulated key mitophagy proteins (‌Pink1‌, ‌Parkin‌, and ‌LC3II‌), indicating that Sirt1 deficiency enhances mitophagy activity (Fig. 7F)‌. Meanwhile, laser scanning confocal microscopy also revealed that high-glucose induces cytoplasmic translocation of endogenous Pink1, which colocalized with Sirt1, suggesting a regulatory role of Sirt1 in Pink1 expression (Fig. 7G).‌

## Discussion

The hepatosomatic index (HSI), recognized as a key indicator for evaluating liver size and overall health in fish [46, 47], showed significant elevation in grouper (*Epinephelus akaara*) [48] and largemouth bass fed HC diet, with similar changes observed in VSI. It was well known that carnivorous fish typically alleviate high glucose loads by enhancing glycolysis (involving hexokinase, PFK-1, and pyruvate kinase) and activating gluconeogenesis [49], this study's PAS staining, electron microscopy, and enzymatic analyses revealed excessive hepatic glycogen accumulation, which may due to dietary carbohydrate levels exceeding metabolic regulatory capacity. When glucose metabolism becomes overloaded, the AMPK/SREBP-1c axis drives hyperactivated lipogenesis, converting excess carbon sources into stored triglycerides [50]. Subsequent cellular experiments revealed a greater accumulation of lipid droplets in cells treated with high-glucose, further corroborating that a high-carbohydrate diet induced an over-accumulation of lipid in the liver. A similar phenomenon was also observed in Nile tilapia (*Oreochromis niloticus*) [51], gibel carp (*Carassius gibelio*) [52], and rainbow trout (*Oncorhynchus mykiss*) [53]. Additionally, prior research on largemouth bass fed an 18% carbohydrate diet demonstrated reduced serum insulin levels, downregulated hepatic *insulin* gene expression, and upregulated insulin resistance-related genes [41], indicating that high-glucose-induced liver injury may correlate with impaired insulin synthesis.

Lipid accumulation is well recognized to be mediated by AMPK and SREBP. The activated form of SREBP-1 is responsible for the upregulation of gene expression related to lipid biosynthesis, leading to an increase in lipid droplet formation and overall lipid content [54]. It has been shown that the high-glucose treatment of hepatocytes suppresses the expression of SREBP-1 target genes by AMPK by repressing its cleavage and translocation intranuclearly [55]. Interestingly, HC diet could increase lipid production by inhibiting AMPK, causing *acc*, *srebp1*, and *fas* to be increased. Additionally, FABP1 functions as a transport agent, a transporter, and a metabolic regulator of fatty acids [56]. This study found that HC diet significantly upregulated *apob*, *apob100* and *fabp1* in largemouth bass that had fatty liver, indicating that it promoted the absorption and transport of fatty acids and the synthesis of triglycerides. Notably, the HC diet exerted limited effects on key genes regulating fatty acid oxidation (*cpt1*, *ppar-α*), indicating that lipid accumulation predominantly originates from enhanced synthesis rather than suppressed breakdown.

Current findings indicated that liver damage and lipid accumulation in largemouth bass can be induced by HC diet. Similar phenomena have been reported in other fish species [57–59]. Liver injury is primarily attributed to the initial toxic effects of excessive lipids [60]. Mounting evidence shows that the accumulation of both cholesterol and triglycerides may result in lipotoxicity [61]. The present study revealed that the HC diet disrupts cholesterol homeostasis by downregulating critical bile acid synthesis genes (*cyp7a1*, *cyp8b1*) and the regulatory gene *shp*. This suppression impedes the conversion of cholesterol to bile acids, causing pathological accumulation of free cholesterol. Beyond that, abnormal accumulation of free cholesterol is believed to compromise the integrity of mitochondrial and endoplasmic reticulum membranes, thereby exacerbating promote mitochondrial oxidative damage and endoplasmic reticulum stress, and finally induce and aggravate liver injury [62]. ‌Although hepatic *hmgcr* (the rate-limiting enzyme for cholesterol synthesis) expression remained unchanged, inhibition of the cholesterol breakdown pathway was still a central contributor to metabolic dysregulation. Similarly, we observed that the HC diet did not alter hepatic *fxr* or *rxrα* mRNA expression, aligning with previous research [63]. This might be because FXR primarily functions in the liver-gut axis, and the HC diet has a less effect on regulating *fxr* expression in the liver.‌

Mitochondria serve as the central hub for glucose and lipid metabolism, functioning as both sensors and executors of metabolic signals [64]. Research indicates that their homeostasis (fusion/fission) directly responds to cellular metabolic demands, while in mammals, disruption of this equilibrium has been identified as a critical factor in high-glucose/high-fat-induced hepatic injury [65, 66]. In fish, it has been reported that high-fat diet in *Pelteobagrus pelteobagrus* can activate mitochondrial biogenesis, promote mitochondrial fusion and induce oxidative stress, consequently leading to increased lipid accumulation [31]. Prolonged consumption of high-carbohydrate diet has been associated with the induction of oxidative stress, which may impair mitochondrial respiratory chain activity in the liver of rainbow trout [67] and *Megalobrama amblycephala* [68]. In this study, HG treatment upregulated the fission protein Drp1 and downregulated the fusion protein Tom20, triggering mitochondrial fragmentation. The fission process was accompanied by a burst of mitoROS, which directly suppresses respiratory chain function. Excessive mitoROS activated the Pink1/Parkin pathway to initiate mitophagy, yet sustained activation exacerbates the cellular energy crisis. Ultimately, the AMPK/ACC/SREBP-1 axis-driven enhanced lipogenesis, cholesterol dysmetabolism, and mitochondrial fission-autophagy cascade collectively drive liver injury.

In mammals, Sirt1 serves as a central hub in nonalcoholic fatty liver disease (NAFLD)/nonalcoholic steatosis (NASH) pathogenesis by integrating the regulation of lipid and glucose metabolism with inflammatory responses [69, 70]. It has reported that Sirt1 suppresses fatty acid and triglyceride synthesis by deacetylating and inhibiting the transcription factor SREBP1c, while concurrently activating the AMPK pathway to promote fatty acid oxidation, thereby alleviating hepatocellular steatosis [71].‌ ‌Furthermore, Sirt1 mitigates hepatic inflammation and fibrosis by inhibiting NF-κB pathway activation, which reduces the release of pro-inflammatory cytokines such as TNF-α and IL-6 [72]. It also enhances antioxidant capacity to counteract oxidative damage characteristic of NASH [73]. In recent years, research has begun to uncover the significant role of Sirt1 in the metabolic regulation of fish species. Resveratrol, known as a Sirt1 activator, demonstrated the ability to alleviate oxidative stress and inflammation in LPS-injected gibel carp by boosting the Sirt1/PGC-1α and Pink1/Parkin pathways [74]. In tilapia fed high-carbohydrate diets, activation of the Sirt1/AMPK signaling pathway effectively alleviated high-glucose-induced metabolic syndrome [59]. Further investigations showed that Sirt1 and FoxO1 work together to manage glucose metabolism in the kidney and intestinal tissues of blunt snout bream under conditions of high-glucose intake [75]. Recent studies also revealed that Sirt1 attenuates hepatic lipotoxicity by suppressing the activation of the JNK signaling pathway [76]. ‌This study demonstrates that high-glucose specifically suppresses *sirt1* expression (but not other sirtuins) and induces its nucleocytoplasmic translocation-a mechanism that corresponds to Xu et al.'s [77] report of nuclear Sirt1 degradation via LC3 binding and the autophagosome-lysosome pathway during aging. In mammals, Sirt1 governs early-stage NAFLD progression through metabolic and inflammatory regulation, whereas the Pink1/Parkin pathway serves as the central executor of mitochondrial quality control. Here, we reveal Sirt1 primarily functions as an energy effector: high glucose-induced nucleocytoplasmic translocation promotes its binding to autophagosomes, precipitating mitochondrial dysfunction and consequent disruption of hepatocellular energy homeostasis. Additionally, the evolutionary conservation of Sirt1 across species, combined with its lineage-specific branching patterns, underscores its central role in metabolic regulation.

## Conclusions

To conclude, our results demonstrate first-time evidence that the AMPK-mediated ACC/SREBP-1 pathway contributes to lipid accumulation in largemouth bass, both in vivo and in vitro. Simultaneously, this study is also the first to establish a primary hepatocyte injury model using high-glucose, confirming its role in mitochondrial damage and glycolipid accumulation. Moreover, we uncovered a correlation between mitophagy and high-glucose treatment, and determined that high-glucose compromises Sirt1's metabolic protective function by suppressing its expression and altering its subcellular localization (nucleus-to-cytoplasm translocation), thereby activating Pink1/Parkin-mediated mitophagy.

## Data Availability

All data generated or analyzed during this study are included.

## Abbreviations

*acadm*: Acyl-CoA dehydrogenase medium
*acc1*: Acetyl-CoA carboxylase 1
AMPK: Adenosine 5 ‘-monophosphate (AMP)-activated protein kinase
*apob*: Apolipoprotein B
*atgl*: Adipose triglyceride lipase
BA: Bile acid
CCK8: Cell counting kit-8
CON: Control
*cpt1*: Carnitine palmitoyltransferase 1
*cyp7a1*: Cholesterol 7-hydroxylase
*cyp8b1*: Sterol 12-hydroxylase
*cytb*: Cytochrome b
EDTA: Ethylenediaminetetraacetic acid
*ef-1α*: Elongation factor 1α
*ennp1*: Phosphodiesterase 1
*fabp1*: Fatty acid binding protein 1
*fas*: Fatty acid synthase
FBS: Fetal bovine serum
*fgfr4*: Fibroblast growth factor receptor 4
FITC: Fluorescein isothiocyanate
FXR: Farnesoid X receptor
*gsy2*: Glycogen synthase 2
HC: High-carbohydrate
HG: High-glucose
HSI: Hepatosomatic index
*hsl*: Hormone-sensitive lipase
Keap1: Kelch-like ECH-associated protein 1
LG: Low glucose
*lpl*: Lipoprotein lipase
MAPK: Microtubule-associated protein kinase
mitoROS: Mitochondrial reactive oxygen species
MMP: Mitochondrial membrane potential
NAFLD: Nonalcoholic fatty liver disease
NASH: Nonalcoholic steatosis
NIH: National institutes of health
*nqo1*: NAD(P)H oxidoreductase 1
*nrf2*: Nuclear factor erythroid 2-related factor 2
PAS: Periodic acid-schiff
PBS: Phosphate-buffered saline
PI: Propidium iodide
*ppar-α*: Peroxisome proliferator-activated receptor alpha
*ppar-γ*: Peroxisome proliferator-activated receptor γ
qRT-PCR: Quantitative real-time PCR
ROS: Reactive oxygen species
*shp*: Small heterodimer partner
*srebp1*: Sterol regulatory element-binding protein 1
TEM: Transmission electron microscopy
VSI: Viscerosomatic index

## References

[1] Li X, Zheng S, Han T, Song F, Wu G. Effects of dietary protein intake on the oxidation of glutamate, glutamine, glucose and palmitate in tissues of largemouth bass (Micropterus salmoides). Amino Acids. 2020;52(11–12):1491–503. 10.1007/s00726-020-02907-3 33161445

[2] Lin S, Shi CM, Mu MM, Chen YJ, Luo L. Effect of high dietary starch levels on growth, hepatic glucose metabolism, oxidative status and immune response of juvenile largemouth bass, Micropterus salmoides. Fish Shellfish Immunol. 2018;78:121–6. 10.1016/j.fsi.2018.04.046 29684600

[3] Xue M, Yao T, Xue M, Francis F, Qin Y, Jia M, et al. Mechanism analysis of metabolic fatty liver on largemouth bass (Micropterus salmoides) based on integrated lipidomics and proteomics. Metabolites. 2022;12(8):759. 36005631 10.3390/metabo12080759 PMC9415018

[4] Zhang Y, Xie SW, Wei HL, Zheng L, Liu ZL, Fang HH, et al. High dietary starch impaired growth performance, liver histology and hepatic glucose metabolism of juvenile largemouth bass, Micropterus salmoides. Aquac Nutr. 2020;26(4):1083–95.

[5] Li X, Zheng S, Ma XT, Song F, Wu G. Effects of dietary starch and lipid levels on the protein retention and growth of largemouth bass (Micropterus salmoides). Amino Acids. 2020;52(6–7):999–1016. 32648068 10.1007/s00726-020-02869-6

[6] Zhang BY, Yang HL, Nie QJ, Zhang Y, Cai GH, Sun YZ. High dietary wheat starch negatively regulated growth performance, glucose and lipid metabolisms, liver and intestinal health of juvenile largemouth bass Micropterus salmoides. Fish Physiol Biochem. 2024;50(2):635–51. 38165563 10.1007/s10695-023-01295-1

[7] Li S, Sang C, Turchini GM, Wang A, Zhang J, Chen N. Starch in aquafeeds: the benefits of a high amylose to amylopectin ratio and resistant starch content in diets for the carnivorous fish, largemouth bass (Micropterus salmoides). Br J Nutr. 2020;124(11):1145–55. 32624026 10.1017/S0007114520002214

[8] Xie S, Xu J, Chen L, Qi Y, Yang H, Tan B. Single-cell transcriptomic analysis revealed the cell population changes and cell-cell communication in the liver of a carnivorous fish in response to high-carbohydrate diet. J Nutr. 2024;154(8):2381–95. 38945299 10.1016/j.tjnut.2024.06.016

[9] Zhou YL, He GL, Jin T, Chen YJ, Dai FY, Luo L, et al. High dietary starch impairs intestinal health and microbiota of largemouth bass, *Micropterus salmoides*. Aquaculture. 2021;534:736261.

[10] Alves A, Pereira RT, Rosa PV. Morphology of the digestive system in carnivorous freshwater Dourado salminus brasiliensis. J Fish Biol. 2021;99(4):1222–35. 34085710 10.1111/jfb.14821

[11] Li S, Wang A, Li Z, Chen YJ, Dai FY, Luo L, et al. Antioxidant defenses and non-specific immunity at enzymatic and transcriptional levels in response to dietary carbohydrate in a typical carnivorous fish, hybrid grouper (Epinephelus fuscoguttatus ♀× E. Lanceolatus ♂). Fish Shellfish Immunol. 2020;100:109–116. 32156583 10.1016/j.fsi.2020.03.015

[12] Bechmann LP, Hannivoort RA, Gerken G, Hotamisligil GS, Trauner M, Canbay A. The interaction of hepatic lipid and glucose metabolism in liver diseases. J Hepatol. 2012;56(4):952–64. 22173168 10.1016/j.jhep.2011.08.025

[13] Xie D, Yang L, Yu R, Chen F, Lu R, Qin C, et al. Effects of dietary carbohydrate and lipid levels on growth and hepatic lipid deposition of juvenile tilapia, Oreochromis niloticus. Aquaculture. 2017;479:696–703.

[14] Li S, Sang C, Zhang J, Li Z, Chen N. Molecular cloning, expression profiling of adipose triglyceride lipase (atgl) and forkhead box o1 (foxo1), and effects of dietary carbohydrate level on their expression in hybrid grouper (Epinephelus fuscoguttatus ♀ × E. Lanceolatus ♂). Aquaculture. 2018;492:103–112.

[15] Cai W, Liang X, Yuan X, Li Z, Chen N. Different strategies of grass carp (Ctenopharyngodon idella) responding to insufficient or excessive dietary carbohydrate. Aquaculture. 2018;497:292–8.

[16] Liang X, Chen P, Wu X, Xin S, Morais S, He M, et al. Effects of high starch and supplementation of an olive extract on the growth performance, hepatic antioxidant capacity and lipid metabolism of largemouth bass (Micropterus salmoides). Antioxidants. 2022;11(3):577. 35326228 10.3390/antiox11030577 PMC8945146

[17] Wang H, He Q, Wang G, Xu X, Hao H. Fxr modulators for enterohepatic and metabolic diseases. Expert Opin Ther Patents. 2018;28(11):765–82. 10.1080/13543776.2018.1527906 30259754

[18] Wang LX, Frey MR, Kohli R. The role of fgf19 and malrd1 in enterohepatic bile acid signaling. Front Endocrinol. 2021;12:799648. 10.3389/fendo.2021.799648 PMC8804323 35116006

[19] Gehart H, Kumpf S, Ittner A, Ricci R. MAPK signaling in cellular metabolism: stress or wellness? Embo Rep. 2010;11(11):834–40. 20930846 10.1038/embor.2010.160 PMC2966959

[20] Deng J, Zhang X, Lin B, Mi H, Zhang L. Excessive dietary soluble arabinoxylan impairs the intestinal physical and immunological barriers via activating MAPK/NF-κB signaling pathway in rainbow trout (Oncorhynchus mykiss). Fish Shellfish Immunol. 2023;141:109041. 37657558 10.1016/j.fsi.2023.109041

[21] Song ZX, Jiang WD, Liu Y, Wu P, Jiang J, Zhou XQ, et al. Dietary zinc deficiency reduced growth performance, intestinal immune and physical barrier functions related to NF-κB, TOR, Nrf2, JNK and MLCK signaling pathway of young grass carp (Ctenopharyngodon idella). Fish Shellfish Immunol. 2017;66:497–523. 28549941 10.1016/j.fsi.2017.05.048

[22] Li X, Cui K, Fang W, Chen Q, Xu D, Mai K, et al. High level of dietary olive oil decreased growth, increased liver lipid deposition and induced inflammation by activating the p38 MAPK and JNK pathways in large yellow croaker (Larimichthys crocea). Fish Shellfish Immunol. 2019;94:157–65. 31465874 10.1016/j.fsi.2019.08.062

[23] Zhang W, Dan Z, Zheng J, Du J, Liu Y, Zhao Z, et al. Optimal dietary lipid levels alleviated adverse effects of high temperature on growth, lipid metabolism, antioxidant and immune responses in juvenile turbot (Scophthalmus maximus l.). Comp Biochem Physiol B-Biochem Mol Biol. 2024;272:110962. 38387739 10.1016/j.cbpb.2024.110962

[24] Li M, Hu FC, Qiao F, Du ZY, Zhang ML. Sodium acetate alleviated high-carbohydrate induced intestinal inflammation by suppressing MAPK and NF-κB signaling pathways in Nile tilapia (Oreochromis niloticus). Fish Shellfish Immunol. 2020;98:758–65. 31730927 10.1016/j.fsi.2019.11.024

[25] Liu H, Chen B, Cao Y, Geng Y, Ouyang P, Chen D, et al. High starch diets attenuate the immune function of Micropterus salmoides immune organs by modulating Keap1/Nrf2 and MAPK signaling pathways. Fish Shellfish Immunol. 2023;142:109079. 37774900 10.1016/j.fsi.2023.109079

[26] Sule R, Condon L, Gomes A. Common feature of pesticides: oxidative stress-the role of oxidative stress in pesticide-induced toxicity. Oxid Med Cell Longev. 2022;2022:5563759. 35096268 10.1155/2022/5563759 PMC8791758

[27] Catalani E, Silvestri F, Bongiorni S, Taddei AR, Fanelli G, Rinalducci S, et al. Retinal damage in a new model of hyperglycemia induced by high-sucrose diets. Pharmacol Res. 2021;166:195488. 10.1016/j.phrs.2021.105488 33582248

[28] Zhang D, Zhao T, Hogstrand C, Ye H, Xu X, Luo Z. Oxidized fish oils increased lipid deposition via oxidative stress-mediated mitochondrial dysfunction and the CREB1-Bcl2-Beclin1 pathway in the liver tissues and hepatocytes of yellow catfish. Food Chem. 2021;360:129814. 34023714 10.1016/j.foodchem.2021.129814

[29] Prisingkorn W, Jakovlić I, Yi S, Deng F, Zhao Y, Wang W. Gene expression patterns indicate that a high-fat-high-carbohydrate diet causes mitochondrial dysfunction in fish. Genome. 2019;62(2):53–67. 30830800 10.1139/gen-2018-0159

[30] Shen HC, Chen ZQ, Chen F, Chen S, Ning LJ, Tian HY, et al. DHA alleviates high-glucose-induced mitochondrial dysfunction in Oreochromis niloticus by inhibiting DRP1-mediated mitochondrial fission. Int J Biol Macromol. 2023;244:125409. 37327936 10.1016/j.ijbiomac.2023.125409

[31] Xu C, Liu WB, Zhang DD, Shi JH, Zhang L, Li XF. Benfotiamine, a lipid-soluble analog of vitamin B1, improves the mitochondrial biogenesis and function in blunt snout bream (Megalobrama amblycephala) fed high-carbohydrate diets by promoting the AMPK/PGC-1β/NRF-1 axis. Front Physiol. 2018;9:1–15. 30233383 10.3389/fphys.2018.01079 PMC6129842

[32] Tian L, Cao W, Yue R, Yuan Y, Guo X, Qin D, et al. Pretreatment with Tilianin improves mitochondrial energy metabolism and oxidative stress in rats with myocardial ischemia/reperfusion injury via AMPK/SIRT1/PGC-1 alpha signaling pathway. J Pharmacol Sci. 2019;139:352–60. 30910451 10.1016/j.jphs.2019.02.008

[33] An Y, Wang B, Wang X, Dong G, Jia J, Yang Q. Sirt1 inhibits chemoresistance and cancer stemness of gastric cancer by initiating an AMPK/FOXO3 positive feedback loop. Cell Death Dis. 2020;11:115. 32051395 10.1038/s41419-020-2308-4 PMC7015918

[34] Li HY, Zheng FC, Zhang YM, Sun JJ, Gao FF, Shi GG. Resveratrol, novel application by preconditioning to attenuate myocardial ischemia/reperfusion injury in mice through regulate AMPK pathway and autophagy level. J Cell Mol Med. 2022;26(15):4216–29. 35791579 10.1111/jcmm.17431 PMC9345293

[35] Wang YY, Wang HM, Zhuo YY, Hu YZ, Zhang ZT, Ye JX, et al. SIRT1 alleviates high-magnitude compression-induced senescence in nucleus pulposus cells via PINK1-dependent mitophagy. Aging (Albany NY). 2020;12(16):16126–41. 32687063 10.18632/aging.103587 PMC7485741

[36] Esfahani M, Rahbar AH, Asl SS, Bashirian S, Moeini ES, Mehri F. The effects of resveratrol on silica-induced lung oxidative stress and inflammation in rat. Saf Health Work. 2023;14(1):118–23. 36941929 10.1016/j.shaw.2023.02.001 PMC10024237

[37] Iside C, Scafuro M, Nebbioso A, Altucci L. SIRT1 activation by natural phytochemicals: an overview. Front Pharmacol. 2020;11:1225. 32848804 10.3389/fphar.2020.01225 PMC7426493

[38] Wang KW, Liu QQ, Zhu J, Deng X, Luo L, Lin SM, et al. Transcriptome analysis provides insights into the molecular mechanism of liver inflammation and apoptosis in juvenile largemouth bass Micropterus salmoides fed low protein high starch diets. Comp Biochem Physiol D-Genomics Proteomics. 2023;45:101047. 10.1016/j.cbd.2022.101047 36508948

[39] Xie Y, Shao X, Zhang P, Zhang H, Yu J, Yao X, et al. High starch induces hematological variations, metabolic changes, oxidative stress, inflammatory responses, and histopathological lesions in largemouth bass Micropterus salmoides). Metabolites. 2024;14(4):236. 38668364 10.3390/metabo14040236 PMC11051861

[40] Shi B, Qian T, Yin Z, Zhang Y, Feng T, Dong Z, et al. Comparing effects of high starch diet or high lipid diet supplemented with different levels of zinc on intestinal barrier and microbe community in largemouth bass Micropterus salmoides. Fish Shellfish Immunol. 2024;154:109911. 39293705 10.1016/j.fsi.2024.109911

[41] Liao ZH, He XS, Chen AQ, Zhong J, Lin SH, Guo YC, et al. Astaxanthin attenuates glucose-induced liver injury in largemouth bass: role of p38MAPK and PI3K/Akt signaling pathways. Cell Biosci. 2024;14(1):122. 39300527 10.1186/s13578-024-01304-7 PMC11414117

[42] Yu LL, Yu HH, Liang XF, Li N, Wang X, Li FH, et al. Dietary butylated hydroxytoluene improves lipid metabolism, antioxidant and anti-apoptotic response of largemouth bass (Micropterus salmoides). Fish Shellfish Immunol. 2018;72:220–9. 29108969 10.1016/j.fsi.2017.10.054

[43] Hauck L, Stanley-Hasnain S, Fung A, Grothe D, Rao V, Mak TW, et al. Cardiac-specific ablation of the e3 ubiquitin ligase mdm2 leads to oxidative stress, broad mitochondrial deficiency and early death. Plos One. 2017;12(12):e0189861. 10.1371/journal.pone.0189861 PMC5739440 29267372

[44] Liu Y, Cao M, Cai Y, Li X, Zhao C, Cui R. Dissecting the role of the FGF19-FGFR4 signaling pathway in cancer development and progression. Front Cell Dev Biol. 2020;8:95. 32154250 10.3389/fcell.2020.00095 PMC7044267

[45] Rodgers J, Lerin C, Haas W, Gygi S, Spiegelman B, Puigserver P. Nutrient control of glucose homeostasis through a complex of PGC-1α and SIRT1. Nature. 2005;434(7029):113–8. 15744310 10.1038/nature03354

[46] Li X, Zheng S, Ma X, Cheng K, Wu G. Use of alternative protein sources for fishmeal replacement in the diet of largemouth bass (Micropterus salmoides). Part I: effects of poultry by-product meal and soybean meal on growth, feed utilization, and health. Amino Acids. 2021;53(1):33–47. 33236255 10.1007/s00726-020-02920-6

[47] Du ZY, Turchini GM. Are we actually measuring growth? -an appeal to use a more comprehensive growth index system for advancing aquaculture research. Rev Aquac. 2022;14(2):525–7.

[48] Wang J, Li X, Han T, Yang Y, Jiang Y, Yang M, et al. Effects of different dietary carbohydrate levels on growth, feed utilization and body composition of juvenile grouper Epinephelus akaara. Aquaculture. 2016;459:143–7.

[49] Li X, Han T, Zheng S, Wu G. Hepatic glucose metabolism and its disorders in fish. Adv Exp Med Biol. 2022;1354:207–36. 34807444 10.1007/978-3-030-85686-1_11

[50] Allende DS, Gawrieh S, Cummings OW, Belt P, Wilson L, Van-Natta M, et al. Glycogenosis is common in nonalcoholic fatty liver disease and is independently associated with ballooning, but lower steatosis and lower fibrosis. Liver Int. 2021;41(5):996–1011. 33354866 10.1111/liv.14773 PMC8052274

[51] Xu R, Li M, Wang T, Zhao YW, Shan CJ, Qiao F, et al. Bacillus amyloliquefaciens ameliorates high-carbohydrate diet-induced metabolic phenotypes by restoration of intestinal acetate-producing bacteria in Nile tilapia. Br J Nutr. 2022;127(5):653–65. 33858522 10.1017/S0007114521001318

[52] Gong Y, Xi L, Liu Y, Lu Q, Zhang Z, Liu H, et al. Sequential activations of CHREBP and srebp1 signals regulate the high-carbohydrate diet-induced hepatic lipid deposition in Gibel carp (Carassius gibelio). Aquac Nutr. 2023;2023:6672985. 37520290 10.1155/2023/6672985 PMC10374375

[53] Yu H, Geng S, Li S, Wang Y, Ren X, Zhong D, et al. The MAPK and AKT/GSK3beta pathways are involved in recombinant proteins fibroblast growth factor 1 (RFGF1 and RFGF1a) improving glycolipid metabolism in Rainbow trout (Oncorhynchus mykiss) fed a high-carbohydrate diet. Anim Nutr. 2024;17:11–24. 38444689 10.1016/j.aninu.2023.10.009 PMC10912841

[54] Walker AK, Jacobs RL, Watts JL, Rottiers V, Jiang K, Finnegan DM, et al. A conserved SREBP-1/phosphatidylcholine feedback circuit regulates lipogenesis in metazoans. Cell. 2011;147(4):840–52. 22035958 10.1016/j.cell.2011.09.045 PMC3384509

[55] Han Y, Hu Z, Cui A, Liu Z, Ma F, Xue Y, et al. Post-translational regulation of lipogenesis via AMPK-dependent phosphorylation of insulin-induced gene. Nat Commun. 2019;10:623. 10.1038/s41467-019-08585-4 PMC6367348 30733434

[56] Ito H, Yamashita H, Nakashima M, Takaki A, Yukawa C, Matsumoto S, et al. Current metabolic status affects urinary liver-type fatty-acid binding protein in normoalbuminuric patients with type 2 diabetes. J Clin Med Res. 2017;9(4):366–373. 10.14740/jocmr2934w PMC5330781 28270898

[57] Ge Y, Zhang L, Chen W, Sun M, Liu W, Li X. Resveratrol modulates the redox response and bile acid metabolism to maintain the cholesterol homeostasis in fish Megalobrama amblycephala offered a high-carbohydrate diet. Antioxidants. 2023;12(1):121. 36670983 10.3390/antiox12010121 PMC9854748

[58] Yu C, Wang L, Cai W, Zhang W, Hu Z, Wang Z, et al. Dietary macroalgae saccharina japonica ameliorates liver injury induced by a high-carbohydrate diet in swamp eel (Monopterus albus). Front Vet Sci. 2022;9:869369. 35774985 10.3389/fvets.2022.869369 PMC9237522

[59] Zhou NN, Wang T, Lin YX, Xu R, Wu HX, Ding FF, et al. Uridine alleviates high-carbohydrate diet-induced metabolic syndromes by activating SIRT1/AMPK signaling pathway and promoting glycogen synthesis in Nile tilapia (Oreochromis niloticus). Anim Nutr. 2023;14:56–66. 37252330 10.1016/j.aninu.2023.03.010 PMC10208930

[60] Marra F, Svegliati-Baroni G. Lipotoxicity and the gut-liver axis in nash pathogenesis. J Hepatol. 2018;68(2):280–95. 29154964 10.1016/j.jhep.2017.11.014

[61] Ioannou GN. The role of cholesterol in the pathogenesis of NASH. Trends Endocrinol Metab. 2016;27(2):84–95. 26703097 10.1016/j.tem.2015.11.008

[62] Zhou M, Liu X, Wu Y, Xiang Q, Yu R. Liver lipidomics analysis revealed the protective mechanism of Zuogui Jiangtang Qinggan formula in type 2 diabetes mellitus with non-alcoholic fatty liver disease. J Ethnopharmacol. 2024;329:118160. 10.1016/j.jep.2024.118160 38588985

[63] Zheng Y, Lu Q, Cao J, Liu Y, Liu H, Jin J, et al. Supplementation of mangiferin to a high-starch diet alleviates hepatic injury and lipid accumulation potentially through modulating cholesterol metabolism in Channel catfish (Ictalurus punctatus). Antioxidants. 2024;13(6):722. 38929161 10.3390/antiox13060722 PMC11200457

[64] Richard J, Youle AW, Van B. Mitochondrial fission, fusion, and stress. Science. 2012;337(6098):1062–5. 22936770 10.1126/science.1219855 PMC4762028

[65] Miettinen T, Bjorklund M. Mitochondrial function and cell size: An allometric relationship. Trends Cell Biology. 2017;27(06):393–402. 10.1016/j.tcb.2017.02.006 28284466

[66] Daiber A, Oelze M, Steven S, Kröller-Schön S, Münzel T. Taking up the cudgels for the traditional reactive oxygen and nitrogen species detection assays and their use in the cardiovascular system. Redox Biol. 2017;12:35–49. 28212522 10.1016/j.redox.2017.02.001 PMC5312509

[67] Eya JC, Yossa R, Perera D, Okubajo O, Gannam A. Combined effects of diets and temperature on mitochondrial function, growth and nutrient efficiency in rainbow trout (*Oncorhynchus mykiss*). Comp Biochem Physiol B Biochem Mol Biol. 2017;212:1–11. 28687361 10.1016/j.cbpb.2017.06.010

[68] Li XF, Wang BK, Xu C, Shi HJ, Zhang L, Liu JD, et al. Regulation of mitochondrial biosynthesis and function by dietary carbohydrate levels and lipid references in juvenile blunt snout bream Megalobrama amblycephala. Comp Biochem Physiol A Mol Integr Physiol. 2019;227:14–24. 30201543 10.1016/j.cbpa.2018.08.008

[69] Tian C, Huang R, Xiang M. SIRT1: Harnessing multiple pathways to hinder NAFLD. Pharmacol Res. 2024;203:107155. 38527697 10.1016/j.phrs.2024.107155

[70] Pinho AV, Mawson A, Gill A, Arshi M, Rooman I. Sirtuin 1 stimulates the proliferation and the expression of glycolysis genes in pancreatic neoplastic lesions. Oncotarget. 2016;7(46):74768–78. 27494892 10.18632/oncotarget.11013 PMC5342700

[71] Hu L, Wang H, Huang L, Zhao Y, Wang J. Crosstalk between autophagy and intracellular radiation response (Review). Int J Oncol. 2016;49:2217–2226. 27748893 10.3892/ijo.2016.3719

[72] Niu B, He K, Li P, Gong J, Zhu X, Ye S, et al. SIRT1 upregulation protects against liver injury induced by a HFD through inhibiting CD36 and the NF‑κB pathway in mouse kupffer cells. Mol Med Rep. 2018;18:1609–15. 10.3892/mmr.2018.9088 PMC6072223 29845302

[73] Corbi G, Conti V, Troisi J, Colucci A, Manzo V, Di P, et al. Cardiac rehabilitation increases SIRT1 activity and β-hydroxybutyrate levels and decreases oxidative stress in patients with HF with preserved ejection fraction. Oxid Med Cell Longev. 2019;2019:7049237. 31885811 10.1155/2019/7049237 PMC6900956

[74] Wu L, Chen Q, Dong B, Geng H, Wang Y, Han D, et al. Resveratrol alleviates lipopolysaccharide-induced liver injury by inducing SIRT1/P62-mediated mitophagy in gibel carp (*Carassius gibelio*). Front Immunol. 2023;14:1177140. 37168854 10.3389/fimmu.2023.1177140 PMC10164966

[75] Huang Y, Wang S, Meng X, Chen N, Li S. Molecular cloning and characterization of Sirtuin 1 and its potential regulation of lipid metabolism and antioxidant response in largemouth bass (Micropterus salmoides). Front Physiol. 2021;12:726877. 34646155 10.3389/fphys.2021.726877 PMC8504536

[76] Mu QQ, Miao LH, Qian LJ, Lin Y, Jiang WQ, Ge XP. Regulation of sirt1 and foxO1 in glucose metabolism of Megalobrama amblycephal. Gene. 2024;903:148172. 38242371 10.1016/j.gene.2024.148172

[77] Xu C, Wang L, Fozouni P, Evjen G, Chandra V, Jiang J, et al. SIRT1 is downregulated by autophagy in senescence and ageing. Nat Cell Biol. 2020;22(10):1170–9. 32989246 10.1038/s41556-020-00579-5 PMC7805578

